# Structural and Mechanistic Characterization of the Flavin-Dependent Monooxygenase and Oxidase Involved in Sorbicillinoid Biosynthesis

**DOI:** 10.1021/acschembio.4c00783

**Published:** 2025-03-07

**Authors:** Gwen Tjallinks, Nicolò Angeleri, Quoc-Thai Nguyen, Barbara Mannucci, Mark Arentshorst, Jaap Visser, Arthur F. J. Ram, Marco W. Fraaije, Andrea Mattevi

**Affiliations:** † Department of Biology and Biotechnology, University of Pavia, Via Adolfo Ferrata 9, Pavia 27100, Italy; ‡ Biomolecular Sciences and Biotechnology Institute, University of Groningen, Nijenborgh 3, Groningen 9747 AG, The Netherlands; § Faculty of Pharmacy, University of Medicine and Pharmacy at Ho Chi Minh City, 41 Dinh Tien Hoang Street, Ben Nghe Ward, District 1, Ho Chi Minh City 70000, Vietnam; ∥ Fungal Genetics and Biotechnology, Institute of Biology Leiden, Leiden University, Sylviusweg 72, Leiden 2333 BE, The Netherlands

## Abstract

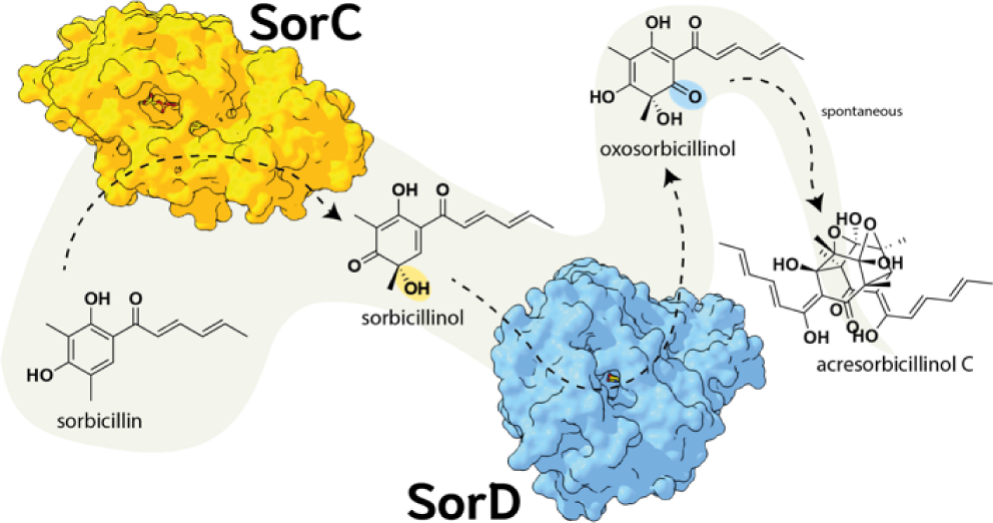

Sorbicillinoids are yellow secondary metabolites synthesized through an elegant combination of enzymatic and spontaneous biochemical processes. The flavin-dependent monooxygenase SorC and oxidase SorD are crucial in this interplay, enabling the generation of a diverse array of functionally complex sorbicillinoids. By solving the crystal structures of SorC and SorD from *Penicillium chrysogenum* with sorbicillin bound in the active site, we describe the catalytically active binding conformations, crucial for attaining enantioselective and stereoselective control in these enzymatic reactions. The structure of SorC was resolved with the cofactor FAD in its *out* state, which allowed us to identify key residues that modulate flavin mobility and other conformational changes. Catalytic residues of SorC were also confirmed by detailed characterization of wild-type and several SorC variants. Meanwhile, using a CRISPR/Cas9-based multicopy-genome integration system, we could heterologously express the flavin-dependent oxidase SorD from *P. chrysogenum* in *Aspergillus niger* with high yields and purity. This allowed us to obtain the crystal structure of SorD with sorbicillin bound in a viable catalytic conformation. Structural analysis of the obtained complex provided insights into the substrate binding pose and highlighted potentially critical active site residues. Ultimately, having both SorC and SorD at our disposal enabled us to investigate their functions and interplays in the biosynthesis of a vast array of functionally complex sorbicillinoids.

## Introduction

Sorbicillinoids are a large family of yellow secondary metabolites produced by various distantly related ascomycetes, including *Trichoderma*(1) and *Penicillium*.^2^ Sorbicillinoids are particularly notable for their complex molecular structures, which often include highly oxygenated bicyclic and tricyclic frameworks.^3^ Sorbicillinoids derive their name from the common structural core sorbicillin, a hexaketide found in many of these metabolites.^4^ The oxidative dearomatization of sorbicillin forms the key monomer sorbicillinol, which characteristically exhibits dual diene and dienophile characteristics. These features allow sorbicillinol to react with itself and other compounds to form various dimeric sorbicillinoids, of which over 90 have been identified.^5−100^ Dimeric sorbicillinoids encompass compounds like bisorbicillinol, generated through an intermolecular Diels–Alder reaction, and exhibit radical-scavenging activity.^9^ Via a Michael addition-like process, other dimers, including bisvertinol and trichodimerol, are formed, which act as potent antagonists of prostaglandin.^10^ As ongoing research continues to explore the full range of these compounds, their diverse biological activities make them promising candidates for drug development and other biotechnological applications.

**Scheme 1:**
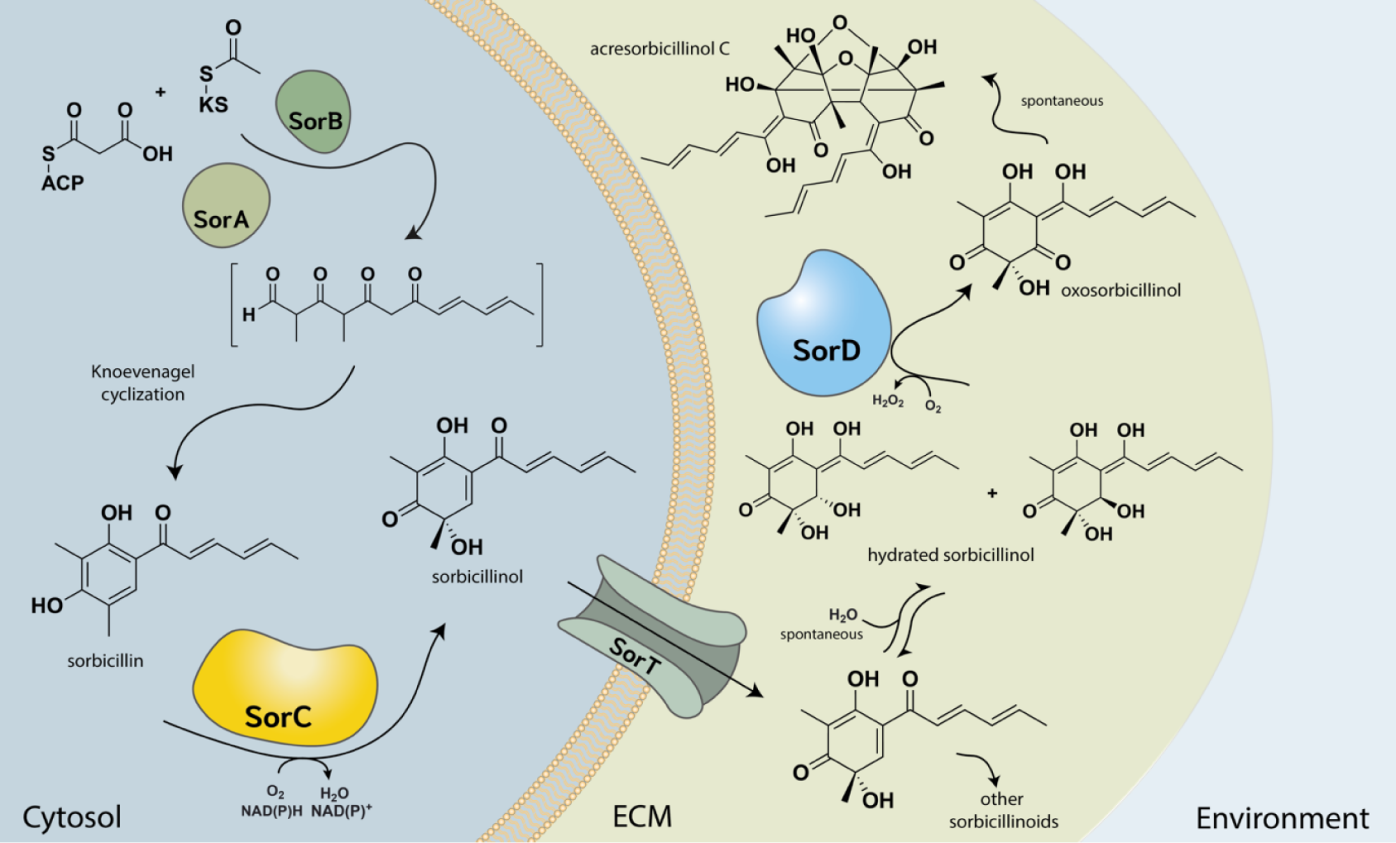
Proposed Model Of Sorbicillin Transport. Sorbicillin is synthesized by polyketide synthases SorA and SorB and is then converted to sorbicillinol by SorC. This compound is transported by the MFS transporter SorT to the extracellular matrix (ECM), where it is oxidized to oxosorbicillinol by SorD. Oxosorbicillinol can sequentially condense with sorbicillin to form acresorbicillinol C. This scheme is based on the results from Duan et al.^22^

The biosynthetic gene clusters responsible for the production of sorbicillinoids have been identified in *Trichoderma reesei* QM6A, *Acremonium chrysogenum* and *Penicillium chrysogenum*.^11−15^ They encode for a highly reducing polyketide synthase (hr-PKS, SorA), a nonreducing polyketide synthase (nr-PKS, SorB), a flavin-dependent monooxygenase (SorC), a flavin-dependent oxidase (SorD), and a major facilitator superfamily membrane transporter (MFS, SorT). SorA and SorB work in tandem to synthesize the hexaketide precursor sorbicillin, while SorC and SorD are involved in the subsequent oxidative processes, including dimerization and oxidation reactions (Scheme 1).^11,12^ Specifically, the monooxygenase SorC catalyzes the oxidative dearomatization of sorbicillin to form (*S*)*-*sorbicillinol.^8,16^ Extensive efforts have been put into analyzing the substrate scope of SorC for its remarkable ability to catalyze chemically challenging regio- and enantioselective reactions that are of high interest in organic chemistry.^17,18^ SorC is part of the group A monooxygenases, of which para-hydroxybenzoate hydroxylase (PHBH; EC 1.14.13.2) functions as a model enzyme. PHBH catalyzes the ortho-hydroxylation reaction of 4-hydroxybenzoate, producing 3,4-dihydroxybenzoate.^19^ SorC also performs an ortho-hydroxylation reaction; however, unlike PHBH, the product sorbicillinol does not undergo rearomatization.^8^ Therefore, sorbicillinol remains reactive for subsequent derivatization reactions, increasing the number of natural products it can biosynthesize.^3,5−7,20,21^ Interestingly, while the examined fungi produce a similar spectrum of sorbicillinoids, their respective SorD enzymes show little sequence homology, suggesting divergent functions in these organisms. Recently, Wang et al.^15^ managed to heterologously express *sorD* from *Acremonium chrysogenum* in *Aspergillus nidulans* to perform *in vitro* enzymatic studies. They concluded that SorD catalyzes the conversion of sorbicillinol to oxosorbicillinol after nonenzymatic hydration with water. Oxosorbicillinol can then spontaneously react with sorbicillinol to form a cage-like bisorbicillinoid called acresorbicillinol C. The same group also identified that the MFS transporter SorT is involved in the transport of sorbicillinol across the cell membrane, where it becomes available for SorD.^22^

In this study, we present the crystal structures of *Penicillium chrysogenum* SorC and SorD with sorbicillin bound to their active sites. Enzymatic and biophysical analyses reveal key residues essential for their catalytic functioning. SorC, a class A monooxygenase, facilitates substrate entry by adopting a flavin *out* conformation, thereby positioning sorbicillin in a pro-*S* configuration ideal for subsequent enantioselective oxidative dearomatization. A recent CRISPR/Cas9-based protein expression system^23^ in *Aspergillus niger* enabled the extracellular production and crystallization of SorD with sorbicillin bound in a catalytically active manner. Ultimately, with both SorC and SorD in hand, we were able to investigate the interplay of these flavin-dependent enzymes.

## Results and Discussion

### Biochemical Characterization of SorC and SorD

**Figure 1:**
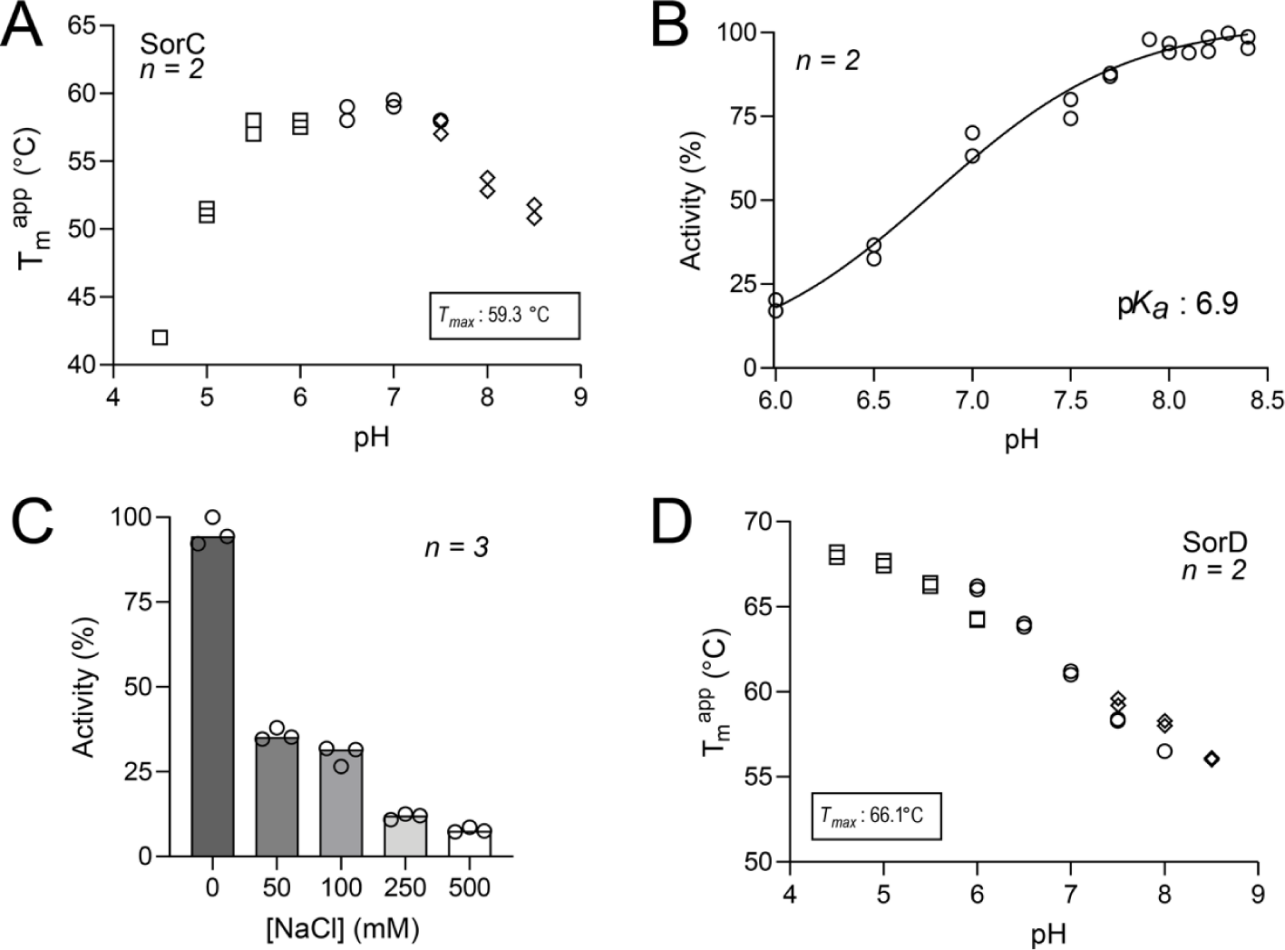
Biochemical characterization of SorC and SorD. (A) Melting temperature profile of SorC at different pH values. Citrate buffer was used for acidic pH (pH 4.5–6.0), KPi buffer for neutral pH (pH 6.5–7.5), and TRIS buffer for basic pH (pH 8–9). (B) Effects of the pH on SorC activity measured by oxygen depletion. (C) Effects of NaCl concentrations on SorC activity measured by substrate depletion at 400 nm in 50 mM KPi (pH 7). (D) Melting temperature profile of SorD at different pH values. Buffer usage was the same as that in (A).

The N-terminally His_6_- and SUMO-tagged SorC was produced in *Escherichia coli* NEB 10-beta cells. One-step Ni-affinity purification yielded 105 mg/L of purified protein, characterized by a distinct yellow color and a typical flavoprotein absorbance spectrum with peaks at 368 and 452 nm (Figures S1 and S2). The thermostability of SorC was assessed across various buffers with pH values ranging from 4.5 to 8.5. The enzyme exhibited the maximum melting temperature of 59 °C in 50 mM KPi pH 6–7 (Figure 1A). The optimal reaction conditions for SorC were investigated by monitoring the oxidative dearomatization at different pH values and temperatures. Mechanistically, the reaction is likely enhanced by deprotonation of the substrate, which increases its nucleophilicity, facilitating subsequent attack on the flavin C4a-hydroperoxy flavin species.^24,25^ The presence of ionizable side chains in the active site, such as the base Glu245, can also contribute to an increase in catalytic efficiency at higher pH. In fact, the optimum activity for the reaction was observed at pH 8, and the curve can be associated with an approximate p*K*_a_ of 6.9 (Figure 1B). Most group A monooxygenases’ catalytic efficiency is reduced by the addition of NaCl due to impeding O_2_ reactivity by potentially binding in the same active site region.^26^ Indeed, the addition of NaCl reduced the catalytic efficiency of SorC (Figure 1C); thus, we decided to perform reactions in buffers without inhibitory anions. Regarding nicotinamide specificity, both NADH and NADPH were accepted as coenzymes, with NADH having a higher overall catalytic efficiency (Figure S3).

The C-terminally His_6_-tagged SorD from *Penicillium chrysogenum* ATCC 48,271 was produced in *Aspergillus niger* following a CRISPR/Cas9-based expression method.^23^ Previous attempts to express soluble SorD in other expression systems were unsuccessful. Sequence analysis of SorD predicts the presence of a signal peptide (using the SignalP 6.0 server from DTU Health Tech),^27^ suggesting that the enzyme is likely localized outside the cell. Likewise, a homologue of SorD from *A. chrysogenum* was recently characterized and shown to be secreted.^22^ The endogenous signal peptide of SorD was used for expression in *A. niger*. The CRISPR/Cas9-based integration system proved to be effective in incorporating up to five copies of the 6xHis-tagged *sorD* gene into the *A. niger* genome. The successful expression and secretion in the medium were confirmed by SDS-PAGE and Western blot analysis (Figure S4). The theoretical molecular weight of SorD is approximately 50 kDa; however, due to several *N*-glycosylation sites, the weight increased to 61 kDa, as confirmed by mass photometry (Figure S5). Ni-affinity purification of His_6_-tagged SorD resulted in 40 mg/L of pure yellow protein, exhibiting a classic flavoprotein absorption spectrum (Figure S6). Moreover, upon testing the thermostability of SorD, we found that the enzyme is more stable under acidic conditions, with the highest melting temperature of 66 °C observed at pH 4.5. This strongly corroborates the extracellular localization of SorD as fungi generally prefer more acidic growth conditions.

### Structural Analysis of SorC

**Figure 2:**
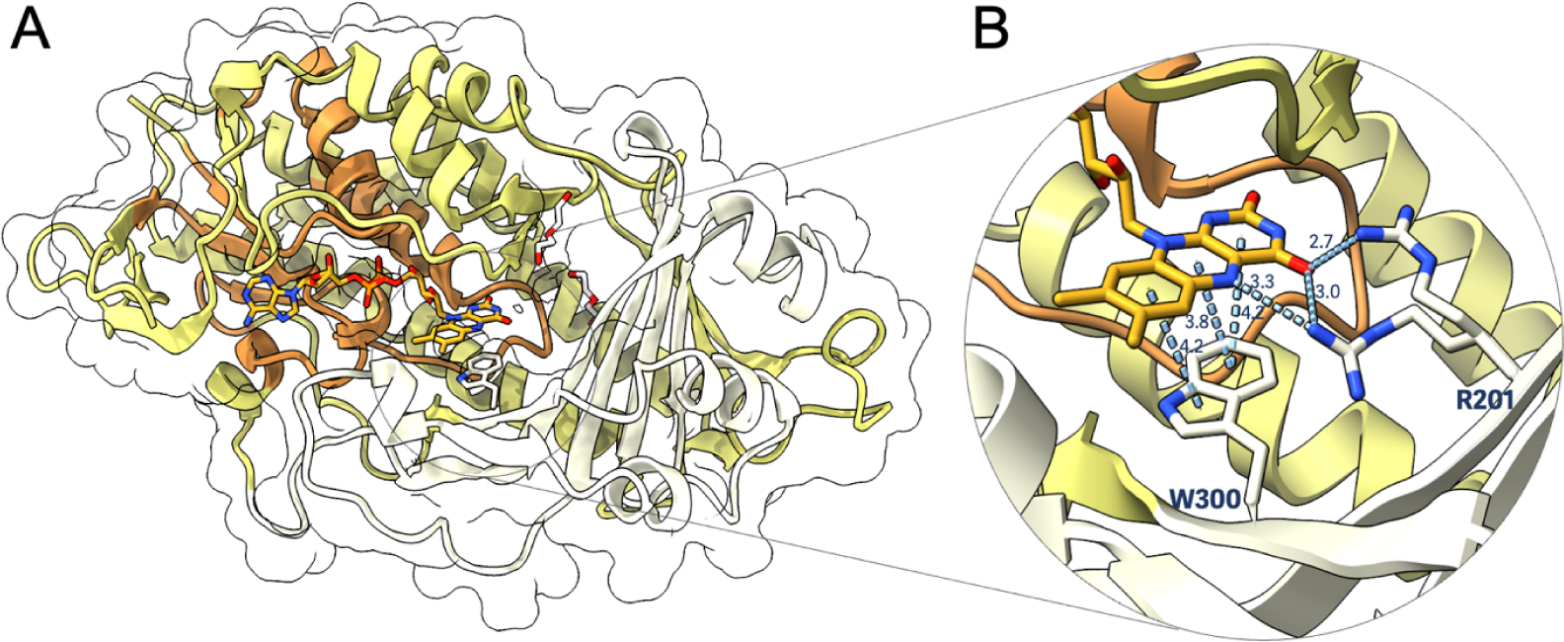
Crystal structure of SorC shown as ribbon diagram. (A) The monomer of SorC consists of the substrate domain in beige, the FAD domain in yellow, and the signature profile of aromatic ring hydroxylase in dark orange. (B) A close-up view of the FAD domain reveals the stabilization of the flavin cofactor in the *out* conformation by π–π stacking interactions between the isoalloxazine ring and residue Trp300, and H-bonding interactions with Arg201 that is in a double conformation. Distances are shown in Å, and atoms are colored following the CPK color code.

SorC crystallized as a monomer, which is consistent with size-exclusion chromatography and mass photometry analyses (Figures S5 and S7). The general structure was solved at 1.4 Å (Table S1), and consists of two domains: a Rossmann fold FAD-binding domain (residues 9–185 and 302–403) containing a binding site for the adenosine diphosphate moiety of FAD, and the catalytic middle domain (residues 186–301) that binds the substrate (Figure 2A). Within the FAD-binding domain, there are specific aromatic ring hydroxylase signatures.

**Figure 3:**
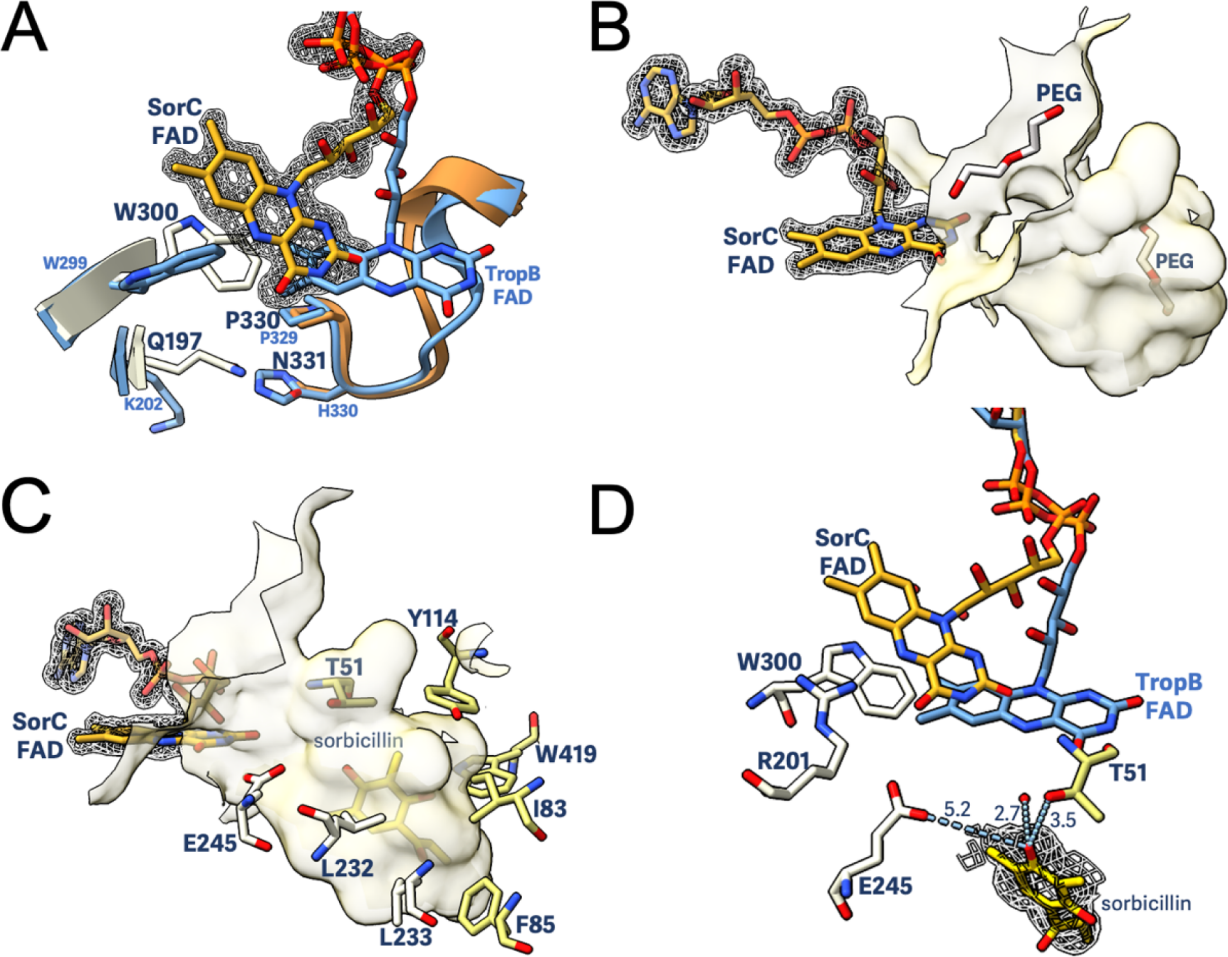
The flavin *out* conformation and substrate binding pocket in SorC. (A) Comparison between SorC with the FAD *out* (in yellow) and TropB (PDB: 6NEV) with the FAD *in* (in blue). Residues Gln197, Trp300, Pro330, and Asn331 are involved in conformational changes, allowing flavin mobility. The FAD polder omit map (in dark gray) was calculated by excluding the cofactor and was contoured at 4 σ. (B) The crystal structure of SorC containing polyethylene glycol (PEG) molecules in the vicinity of the active site pocket. (C) Residues lining the active site are involved in substrate coordination. (D) The electron density shown for sorbicillin bound in the pro-*S* conformation being held in place by Thr51, Glu245, and a water molecule. The FAD from TropB is overlapped with SorC to visualize the positioning with regard to the C4a atom of the flavin cofactor. Distances are shown in Å and atoms are colored following the CPK color code. The sorbicillin polder omit map (in dark gray) was calculated by excluding the ligand and was contoured at 3 σ. The three terminal C atoms of sorbicillin’s alkyl chain in SorC structure were disordered and were not included in the refined model.

To minimize unwanted NAD(P)H oxidase activity, group A monooxygenases employ a nuanced mechanism for recognizing substrates, coenzymes, and oxygen.^28,29^ These monooxygenases have a mobile FAD cofactor that engages distinct sites for flavin reduction and substrate hydroxylation, enabled by several rapid conformational changes to facilitate catalysis.^30^ Analysis of the environment around FAD revealed that the cofactor is in the *out* conformation. Trp300, located on the *re*-face of FAD, forms π–π stacking interactions with the isoalloxazine ring, thereby stabilizing this *out* conformation (Figures 2B and S8A). Furthermore, a flexible Arg201 residue provides additional hydrogen-bonding interactions with the O4 and N5 of the isoalloxazine ring. Another crucial residue likely involved in the conformational change necessary for flavin reduction by NAD(P)H and for the hydroxylation reaction is Pro330. This residue is conserved across all known PHBHs and contributes to local rigidity around a loop close to the flavin (Figure 3A).^31,32^ By superimposing SorC’s structure onto that of PHBH-type monooxygenase TropB (37% sequence identity), which features the FAD *in* conformation, it is evident that this loop adopts a different pose for the latter, allowing the FAD to slide *in*. Moreover, the conserved Trp side-chain in TropB (Trp299) is tilted 90°, thus disrupting the π–π stacking interactions with the isoalloxazine ring. In SorC, the residue Asn331 promotes the FAD *out* conformational change by forming H-bonding interactions with Gln197. In the case of TropB, the equivalent Lys202 is pointing away from His330.

In class A flavin-dependent monooxygenases, substrate binding generally initiates flavin reduction by promoting the *out* conformation of the flavin.^33−35^ The crystal structure of SorC without sorbicillin shows that the oxidized flavin is already in the *out* conformation. This allows a passage for sorbicillin to enter into the active site. In fact, two poly(ethylene glycol) molecules reside in or near the entry of the active site of SorC (Figure 3B). The *out* positioning of the flavin also generates a cavity close to the cofactor that provides space for the nicotinamide to enter and allow reduction of FAD (Figure S8B,C). Despite extensive experimentation (soaking using excess NAD(P)H or sodium dithionite always in the presence of substrate), a SorC structure with the flavin in the *in* conformation could not be obtained (Figure S8A). The same has been observed for many other group A monooxygenases, including MHBH,^36^ PhzS,^37^ RdmE,^38^ Tet(51),^39^ Tet(56),^39^ and PhqK.^40^ Moreover, we did not observe any electron density close to the FAD-binding domain where NAD(P)H is supposed to bind during soaking or cocrystallization with NAD(P)H. It is known that group A monooxygenases exhibit less tight binding of the nicotinamide coenzyme, leading to the immediate release of NAD(P)^+^ once the flavin is reduced.^41^ This transient interaction might explain why it has been challenging to capture NAD(P)(H) in the crystal structure of SorC.

The map of the SorC-FAD-sorbicillin complex obtained by cocrystallization of SorC with sorbicillin resulted in a new electron density in the active site (Figure 3C,D). Binding between SorC and sorbicillin has also been confirmed using microscale thermophoresis, with a *K*_D_ of 940 ± 93 nM for the substrate (Figure S10). Sorbicillin is embedded in a relatively hydrophobic cavity consisting of residues Ile83, Phe85, Leu223, Leu232, and Trp419, with a few polar anchoring residues, including Thr51, Tyr114, and Glu245 (Figures 3C and S9). The polar residues Thr51 and Glu245, along with a water molecule, secure sorbicillin in the observed orientation by hydrogen-bonding interactions (Figure 3D). Once the flavin is reduced, it can swing *in* and react with oxygen to form the C4a-hydroperoxy flavin species, and come into the proximity of the C5 methyl group of sorbicillin. Overlapping the structure with the *in* conformation of TropB, its FAD cofactor clearly shows the proximity of the substrate to the C4a atom of FAD, demonstrating that this is the catalytically active binding pose for hydroxylation, generating the (*S*)-hydroxylated sorbicillinol. Moreover, the pocket is rather narrow, thereby forcing the aromatic ring to orient in a perpendicular position with respect to the isoalloxazine ring.

### Crucial Active Site Residues in SorC

**Figure 4:**
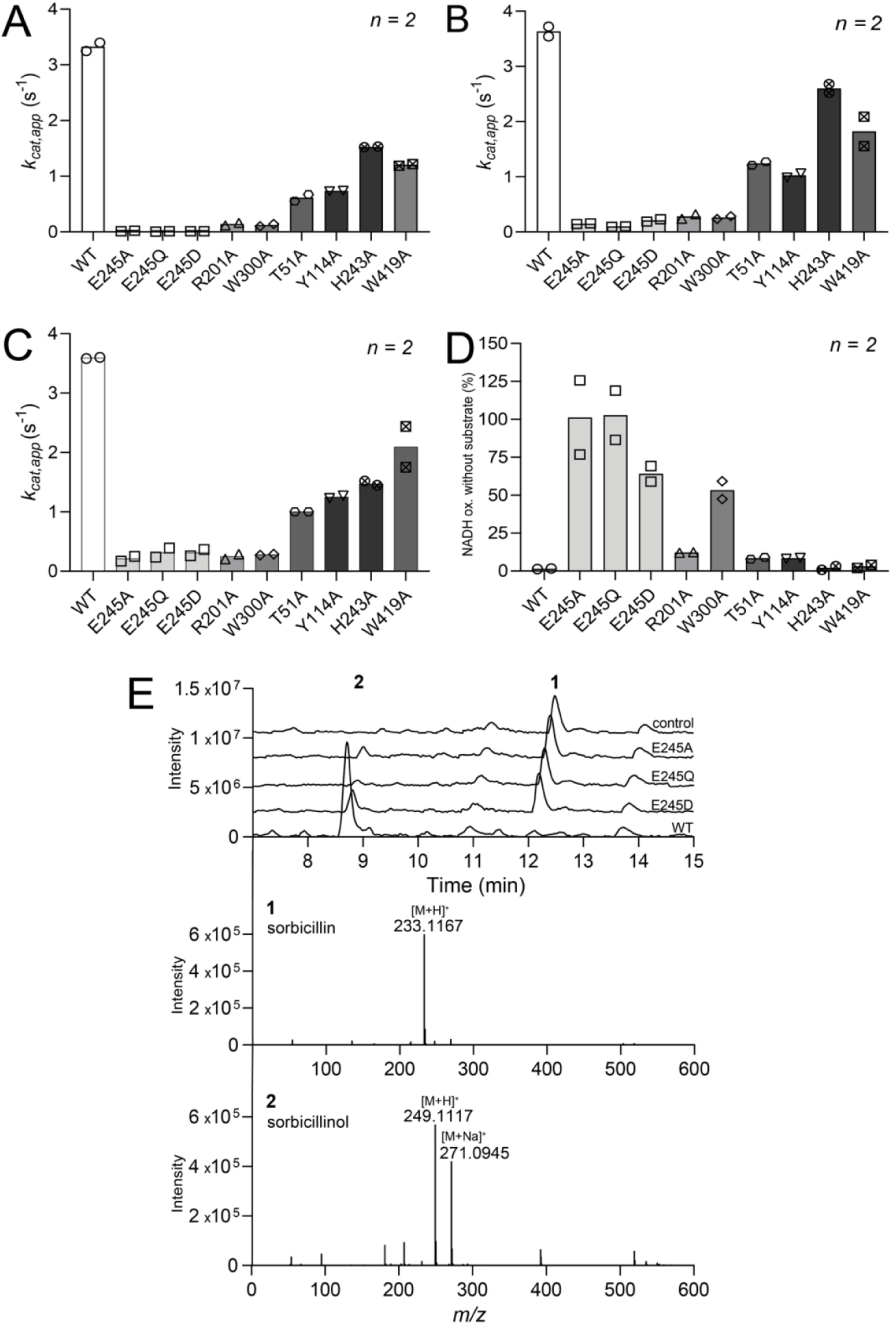
Characterization of wild-type and SorC mutants. Activity by measuring (A) sorbicillin depletion at 400 nm, (B) NADH depletion at 340 nm, and (C) oxygen depletion using the oxygraph. (D) NADH oxidation without the addition of sorbicillin quantified as the percentage of the NADH depletion activities with the addition of sorbicillin (B). Activity values are tabulated in Table S2 including estimated uncoupling percentages. (E) UPLC/HRMS analysis of wild-type and Glu245 SorC mutants for the conversion of sorbicillin (**1**) to sorbicillinol (**2**). The XICs chromatograms were extracted at *m*/*z* 249.1117 for **2** (retention time 8.7 min) and at *m*/*z* 233.1167 for substrate **1** (retention time 12.1 min). The measured *m*/*z* values of the [M + H]^+^ molecular ions of **1** and **2** were detected with an error of −2.1 and −1.6 ppm, respectively. The reaction was performed using 2 mM sorbicillin, 2 μM SorC, and 4 mM NADH in 50 mM KPi (pH 8) at 30 °C for 1 h.

The structure of SorC in complex with its substrate sorbicillin enabled us to identify crucial active site residues. SorC belongs to the group A flavin monooxygenases that typically catalyze regioselective ortho- and para-hydroxylation of phenolics.^42^ Hydroxylation generally occurs through an electrophilic aromatic substitution reaction, where the flavin C4a-hydroperoxy flavin species serves as an electrophile and the substrate containing a hydroxyl (or amino) activating group as nucleophile. Therefore, a common first step in class A flavin-dependent monooxygenases is the deprotonation of the substrate to enhance its nucleophilicity and subsequent attack on the C4a-hydroperoxy flavin.^43^ We hypothesized that residue Glu245 acts as a catalytic base in SorC that facilitates the deprotonation of the C4 hydroxyl group in sorbicillin (Figure 3D). SorC shares relatively low sequence identity (<40%) with other class A flavin-dependent monooxygenases (e.g., TropB, AfoD, and AzaH). In these monooxygenases, Glu245 is not conserved. Instead, they typically contain a hydrophobic residue such as Val or Ile at this position. Nevertheless, our structural data strongly suggested that Glu245 functions as a catalytic base. In fact, mutating Glu245 to Ala virtually abolished the hydroxylation activity (turnover number (*v*/*E*): 0.01 s^–1^, Figure 4A–C). The activity observed at 340 nm was for >99% attributed to NADH oxidation with no substrate conversion (Figure 4D).

To further confirm the role of Glu245 as the catalytic base, we carried out a more conservative change by mutating it to amino acids with a similar charge (Asp) or uncharged with the same length (Gln). The uncharged Glu245Gln variant had an identical loss of activity compared to Glu245Ala (*v*/*E*: 0.01 s^–1^), while the Glu245Asp SorC mutant showed slightly higher activity (*v*/*E*: 0.03 s^–1^) (Figure 4A–C). Conversion experiments were verified by UHPLC/HRMS and confirmed the kinetic data (Figure 4E). Despite the lack of the proposed catalytic base, a slight amount of product formation was still observed in the reactions catalyzed by all three Glu245 mutants. This result can partly be explained by the p*K*_a_ of the substrate: sorbicillin exists either in the protonated or the deprotonated state depending on its p*K*_a_. Since the phenolate form of sorbicillin absorbs at 400 nm, whereas the protonated form at 340 nm, the protonation state could be measured using UV–vis absorbance (Figure S11). The p*K*_a_ of sorbicillin was experimentally determined to be around 7.3, indicating that the majority of sorbicillin exists in its anionic form at pH 8, which accelerates the reaction. Furthermore, it suggests that the presence of a catalytic base might not be strictly necessary for the reaction to proceed, but it can markedly enhance the reaction speed. We conclude that the negative electrostatic charge of Glu245 is likely essential for acting as a catalytic base.

Other amino acids involved in the binding of the substrate in the active site of SorC that possess relevant functional groups are Thr51, Tyr114, His243, Arg201, Trp300, and Trp419. In particular, Arg201 and Trp300 are conserved residues in other class A flavin-dependent monooxygenases.^44^ In TropB, Arg201 was shown to be involved in substrate binding.^45^ For the classic PHBH, Arg220 (corresponding to Arg201 in SorC) was shown to interact with the cofactor by providing a positively charged environment around the pyrimidine portion of the isoalloxazine ring that can enforce the stability of the flavin alkoxide leaving group during hydroxylation.^35,46^ Moreover, an Arg220Gln PHBH mutant was shown to cause global changes in the enzyme’s backbone, allowing for the stabilization of an *open* state rather than the more common *in* and *out* states in the wild-type enzyme.^47,48^ Furthermore, Moran et al.^34^ proved that an Arg220Lys mutation in PHBH stabilized the flavin *out* conformation and that this small perturbation decreased its catalytic efficiency. Inspection of the SorC crystal structure reveals that Arg201 is in the proximity of the FAD cofactor, indicating that it might play a role in regulating the dynamics of the flavin. Mutating this residue to alanine rendered SorC almost completely inactive (Figure 4A–C). This mutation potentially disrupts the stabilization of the alkoxide leaving group during heterolytic fission of the peroxide bond, since binding with sorbicillin was retained, though with a higher *K*_D_ of 12.6 ± 9.7 μM (Figure S10).

The second conserved amino acid is Trp300, which has previously been shown as crucial for the *in*/*out* movement of FAD by π–π stacking with the isoalloxazine ring (Figure 3D).^49^ Mutating this residue to alanine in SorC led to a drastic reduction of sorbicillin conversion, while the enzyme retained NADH oxidation activity (Figure 4A–D). Additionally, the Trp300Ala mutation rendered SorC highly unstable; the mutant quickly precipitated over time, indicating that Trp300 is also a residue necessary for structural stability, as confirmed by the low *T*_m_ of this mutant (Figures S2 and S12).

Mutating the remaining residues Thr51, Tyr114, His243, and Trp419 to alanine reduced the catalytic activity to various extents; however, the mutations never fully inactivated the enzyme (Figure 4A–C). Nevertheless, some mutations (such as Thr51Ala) drastically impaired the stability of the protein, implying their essential roles in proper folding. Further studies will be needed to evaluate the effect of amino acids in the active site that steer the enantioselectivity and stereoselectivity of sorbicillinol biosynthesis.

### Structural Analysis of SorD

**Figure 5:**
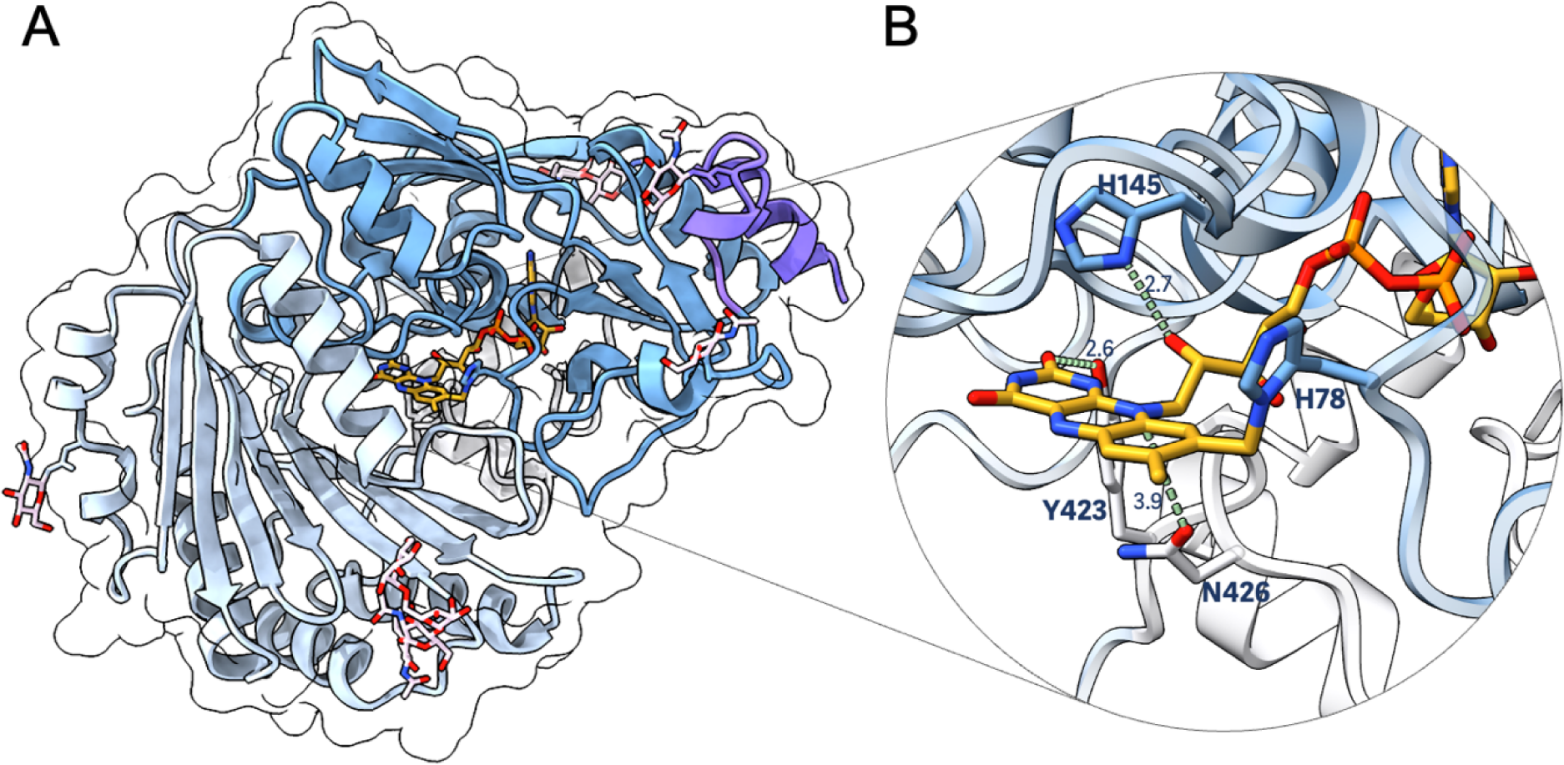
Crystal structure of SorD shown as ribbon diagram. (A) The monomer of SorD contains the substrate domain in light blue, the FAD domain in dark blue, the berberine bridge enzyme domain in white, and N-terminal signal peptide in violet. The glycosylation sites are shown as stick representation. (B) The flavin covalently bound to His78 and made H-bonding interactions with Tyr423, His145, and Asn426. Distances are shown in Å and atoms are colored following the CPK color code.

SorD crystallized as a monomer, consistent with size-exclusion chromatography and mass photometry (Figures S5 and S13). SorD belongs to the vanillyl-alcohol oxidase/*p*-cresol methylhydroxylase (VAO/PCMH) family and more specifically to the berberine bridge enzyme (BBE) subfamily.^50−52^ As with other oxidases from the VAO/PCMH family, the general structure of SorD contains two domains (1.55 Å, Table S1): the N-terminal FAD domain (residues 45–212) and the C-terminal substrate-binding domain (residues 213–435). SorD also comprises a short C-terminal BBE domain (residues 436–468, Figure 5A). The structure contains the partial remains of the N-terminal signal peptide (residues 5–22), and five *N*-glycosylation sites were identified (Asn18, Asn29, Asn174, Asn279, and Asn351).

**Figure 6:**
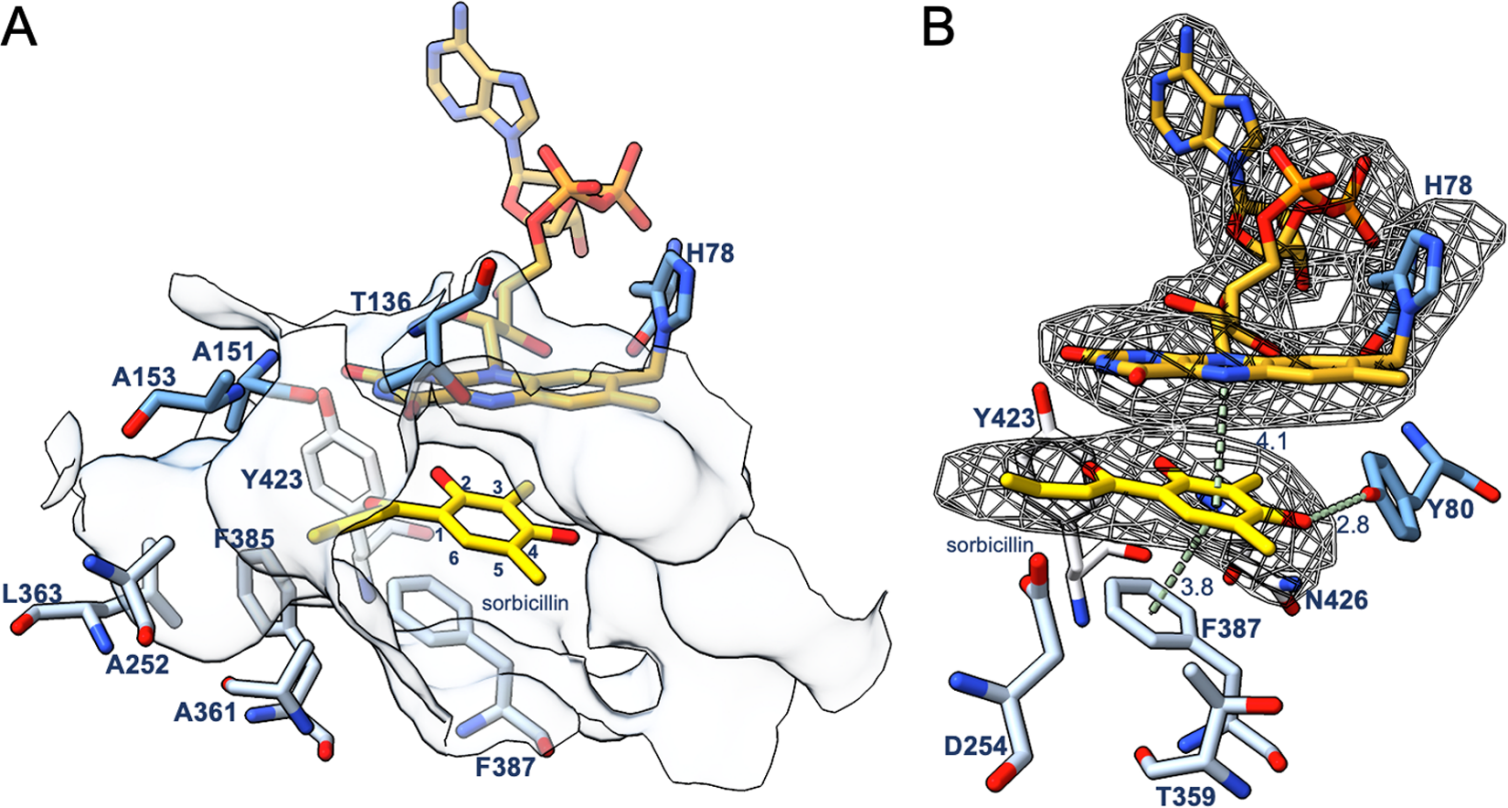
Active site residues involved in sorbicillin binding in chain B of SorD. (A) The hydrophobic active-site pocket surrounding the alkyl chain of sorbicillin in SorD. (B) The stacking of the benzene ring of sorbicillin between Phe387 and the FAD cofactor, and H-bonding interaction with Tyr80. The electron density is displayed for sorbicillin and the His78-linked FAD in chain B. The terminal two carbon atoms from the alkyl chain of sorbicillin are disordered and therefore could not be confidently modeled. Distances are shown in Å and atoms are colored following the CPK color code. The sorbicillin polder omit map was calculated by excluding the ligand and was contoured at 5 σ. The FAD and His78 polder omit map were calculated by excluding the cofactor and residue, and was contoured at 3.5 σ.

Several members of the BBE subfamily are characterized by (bi)covalent incorporation of the flavin cofactor. This feature was also found in SorD, where the FAD is covalently attached to residue His78 (Figure 5B).^53^ BBE-like oxidases are often involved in natural product biosynthesis and polysaccharide oxidation. Covalent tethering of the flavin cofactor in BBE-like oxidases allows for a rather open active site that facilitates the entry of such bulky substrates.^54,55^ We also obtained the structure of SorD with sorbicillin bound at 3.0 Å (Table S1). Even though sorbicillin is not the actual substrate — it lacks the hydroxyl group installed by SorC at the C5 position and the additional hydroxyl group from spontaneous water addition at the C6 position (Scheme 1) — it does mimic hydrated sorbicillinol and binds on the *si*-face near the N5 locus of FAD in both monomers present in the asymmetric units. Binding analysis using microscale thermophoresis also showed that SorD could bind to sorbicillin with a *K*_D_ of 1.2 ± 1.6 μM (Figure S10). The electron density was most pronounced in chain B and gave an indication of the preferred binding manner, where the alkyl chain points toward a smaller side pocket lined by hydrophobic residues Ala151, Ala153, Ala252, Ala361, Leu363, Phe385, and Tyr423 (Figure 6A,B). The terminal atoms would fit perfectly in this hydrophobic region, potentially steering the substrate into the correct position. Residue Phe387, located on the *si*-face of FAD, is important in sandwiching the sorbicillin ring between its own aromatic side-chain and the isoalloxazine ring of FAD by π–π stacking interactions (Figures 6B and S14). Other polar residues present on the *si*-face of the FAD in SorD include Tyr80, Asn426, Asp254, and Thr359. The C4–OH of sorbicillin can bond with Tyr80, and its C2–OH can bond with the pyrimidine-2,4-dione part of the FAD.

The critical observation is that the C6 carbon of bound sorbicillin is in perfect alignment with flavin N5, should hydrated sorbicillinol be placed in the same position (Figure 6A,B). Specifically, in case the hydrated sorbicillinol were present instead of sorbicillin, there would be two additional hydroxyl groups. The first one at C5 would point with its (*S*)-configuration toward residue Thr136 and could allow H-bonding interactions (Figure 6A). The second hydroxyl group at the C6 is added nonenzymatically (Scheme 1) and could therefore be placed both anti- or syn- with reference to the C5–OH. In this binding orientation and assuming an anti-addition, the 6-hydrogen would point toward the flavin N5 atom for hydride transfer, whereas the 6-OH group would be oriented toward Asp254 and Thr359 (Figure 6B). The former two residues might function, directly or indirectly through water-mediated interactions, as a catalytic base that promotes alcohol oxidation.

### Interaction of SorC and SorD

UHPLC/HRMS analysis showed that acresorbicillinol C was produced when SorD was added to the reaction of SorC with sorbicillin and NADH (Figure S15). This result confirmed the activity of SorD, as also recently demonstrated by Wang et al.^15^ We did not see any interactions between the two enzymes (Figure S5). Moreover, no significant change in catalytic efficiency was observed for SorC by spectroscopy when SorD was added to the reaction (Figure S16). This indicated that there are no strong protein–protein interactions, which is in agreement with their distant compartmentalization in the cell. A recent publication from Duan et al.^22^ showed, by colocalization studies in *A. chrysogenum*, that *Ac*SorD was localized outside of the cell and SorC was localized intracellularly. The MFS transporter SorT, embedded in the cell membrane, manages the transport of sorbicillinol across the membrane outside the cell. The compartmentalized biosynthesis of sorbicillinoids could be a potential strategy of fungi to create a more unidirectional efflux of metabolites. In particular, considering the spontaneous chemical transformations of the key monomer sorbicillinol, it might be favorable to promote its export by employing a transporter. The oxidase SorD can then use hydrated sorbicillinol and oxidize it to oxosorbicillinol. The reason why an oxidase is preferred over a short-chain dehydrogenase in this scenario might be attributed to irreversibility: using oxygen as an electron acceptor practically renders the reaction irreversible.

## Supporting Information Available

Detailed materials and methods for strain construction, cloning, mutagenesis, protein expression, protein purification, protein crystallization, biochemical assays, UHPLC/HRMS analysis, and extended experimental data (**Supporting Tables S1–S4 and Supporting Figures S1–S21**). The crystal structures of SorC with and without sorbicillin have been deposited in the Protein Data Bank with accession codes 9H8Z and 9H8M. The crystal structures of SorD, with and without sorbicillin, have been deposited in the Protein Data Bank with accession codes 9H8U and 9H92 (PDF)
(XLSX)

## Abbreviations

FAD: flavin adenine dinucleotide
PHBH: para-hydroxybenzoate hydroxylase
MFS: major facilitator superfamily
VAO/PCMH: vanillyl-alcohol oxidase/*p*-cresol methylhydroxylase
ECM: extracellular matrix
UHPLC/HRMS: ultrahigh-performance liquid chromatography coupled to high-resolution mass spectrometry
NAD(P)H/NAD(P)^+^: nicotinamide adenine dinucleotide (phosphate)
CRISPR: clustered regularly interspaced short palindromic repeats
SUMO: small ubiquitin-related modifier
SDS-PAGE: sodium dodecyl-sulfate polyacrylamide gel electrophoresis
PDB: Protein Data Bank
ESRF: European Synchrotron Radiation Facility
