# Supplementary material for: Structural and Mechanistic Characterization of the Flavin-Dependent Monooxygenase and Oxidase Involved in Sorbicillinoid Biosynthesis
Source: ACS Chem Biol. 2025 Mar 7;20(3):646–55. doi: 10.1021/acschembio.4c00783 (PMC11934079; doi:10.1021/acschembio.4c00783)
Supplement: Supplementary file 1 — cb4c00783_si_001.pdf [file cb4c00783_si_001.pdf]

SUPPLEMENTARY INFORMATION

Structural and Mechanistic Characterization of the Flavin-Dependent Monooxygenase and Oxidase Involved in Sorbicillinoid Biosynthesis

Gwen Tjallinks[1,2], Nicolò Angeleri[1], Quoc-Thai Nguyen[3], Barbara Mannucci[4], Mark Arentshorst[5], Jaap Visser[5], Arthur F. J. Ram[5], Marco W. Fraaije[2*] and Andrea Mattevi[1*].

[1]Department of Biology and Biotechnology, University of Pavia, Via Adolfo Ferrata 9, 27100 Pavia, Italy.
[2]Biomolecular Sciences and Biotechnology Institute, University of Groningen, Nijenborgh 3, 9747 AG Groningen, The Netherlands.
[3]Faculty of Pharmacy, University of Medicine and Pharmacy at Ho Chi Minh City, 41 Dinh Tien Hoang Street, Ben Nghe Ward, District 1, Ho Chi Minh City 70000, Vietnam.
[4]Centro Grandi Strumenti, University of Pavia, Via Bassi 21, 27100, Pavia, Italy
[5]Fungal Genetics and Biotechnology, Institute of Biology Leiden, Leiden University, Sylviusweg 72, 2333 BE Leiden, The Netherlands.

Emails: m.w.fraaije@rug.nl, andrea.mattevi@unipv.it

Table of Contents

1. Methods 4

Chemicals. 4

Cloning, Expression and Purification of SorD. 4

Cloning, Transformation and Mutagenesis of SorC. 5

Expression and Purification of SorC. 5

Protein Crystallization, Structure Determination, and Analysis (SorC). 6

Protein Crystallization, Structure Determination, and Analysis (SorD). 7

Thermostability Analyses. 7

Mass Photometry Experiments. 8

Effect of pH, NaCl and Temperature on SorC. 8

Micro-Scale Thermophoresis. 9

pK_a Determination of Sorbicillin. 9

Extinction Coefficient Determination of Sorbicillin. 9

Steady-State Kinetics. 9

Liquid Chromatography – Mass Spectroscopy Experiments. 10

2. Supplementary tables 12

Table S1. Crystallographic data collection and refinement statistics. 12

Table S2. Activity data. 13

Table S3. Primers used in this study. 13

Table S4. Fasta of native SorC and SorD. 14

3. Supplementary figures 15

Figure S1. Uncropped SDS-PAGE gels of purified His_6-SUMO-tagged SorC wild-type (WT) and mutants. 15

Figure S2. UV-vis absorbance spectrum of purified SorC. 16

Figure S3. Dependence of initial rates on substrate concentrations for wild-type SorC. 17

Figure S4. Different number of insertions of *sorD* into the genome of *Aspergillus niger*. 17

Figure S5. Mass photometry analysis. 18

Figure S6. UV-vis absorbance spectrum of purified SorD. 18

Figure S7. Size-exclusion chromatography of SorC. 19

Figure S8. The flavin positioning in SorC. 19

Figure S9. The interactions between SorC and sorbicillin. 20

Figure S10. Substrate binding to SorC and SorD. 21

Figure S11. pKa determination of sorbicillin 22

Figure S12. The melting temperatures of wild-type SorC, SorC mutants and SorD. 22

Figure S13. Size-exclusion chromatography of SorD. 23

Figure S14. The interactions between SorD and sorbicillin. 23

Figure S15. The LC-MS chromatogram of SorC with and without SorD. 24

Figure S16. The difference in sorbicillin conversion. 25
Figure S17. Crystals of SorC and SorD. 25
Figure S18. The activity of wild-type SorC toward sorbicillin at different temperatures........ 25
Figure S19. The extinction coefficient at 400 nm of sorbicillin 26
Figure S20. The LC-MS chromatogram of sorbicillin conversion by SorC over time. 26
Figure S21. ^{1}H-NMR spectra of sorbicillin........ 27
4. References 28

1. Methods

Chemicals. All commercial chemicals for assays were purchased from Sigma-Aldrich, except for NaOH, MeOH, HCl, K_2HPO_4 and KH_2PO_4 that were purchased from Carlo Erba Reagents. L-arabinose was purchased from Ne-Biotech. NaCl was purchased from VWR Chemicals. PEG10000 was purchased from Fluka. Acetonitrile Ultra LC and Water Ultra LC were purchased from Romil. Bacto Yeast Extract, Bacto Agar and Bacto Tryptone were purchased from Thermo Fisher Scientific. Sorbicillin was purchased from WuXi App. Tech. The purity (HPLC at 220 nm) of sorbicillin was 99%.

Cloning, Expression and Purification of SorD. A recently developed CRISPR/Cas9-based expression method was utilized to establish production of C-terminally His_6-tagged SorD.[1] The *sorD* gene (1416 bp) was amplified from *P. chrysogenum* ATCC 48271 genomic DNA using primers Pc_sorD_P1f and Pc_sorD_P2r (**Table S3-S4**). The predicted signal sequence of 23 amino acids from *P. chrysogenum* ATCC 48271 was employed for the expression in *A. niger*. Primer Pc_sorD_P2r also includes the sequence to include a His_6-Tag at the C-terminus of the protein. The Pc_sorD_P1f and Pc_sorD_P2r primers also include sequences overlapping with PCR amplified fragments consisting of the *glaA* promoter sequence (*PglaA)* and *glaA* terminator sequence (*TglaA*). Primers and conditions for amplification of the *PglaA* and *TglaA* sequences and the fusion PCR are described previously[1]. The *PglaA-sorD-6xHis-TglaA* PCR fragment (2.6 kb) was cloned in pJet1.2 and verified by sequencing. CRISPR/Cas9-mediated targeted integration of the *PglaA-sorD-6xHis-TglaA* construct at predetermined chromosomal loci in the genome of *A. niger* strain MA966.2 was performed as described[1]. In a first transformation, three *PglaA-sorD-6xHis-TglaA* copies were integrated to give strain MA1024.5, following by a second transformation to integrate two additional copies to increase SorD production. Integration of the *PglaA-sorD-6xHis-TglaA* constructs in the genome at various loci was confirmed by diagnostic PCR as described[1]. High-level production (40 mg/L) was achieved in the *Aspergillus niger* strain carrying five chromosomal integrations of the *sorD* gene from *Penicillium chrysogenum* ATCC 48271. 100 mL of complete medium (CM) with freshly harvested spores (1 x 10^8 spores) was utilized to initiate 300 mL Erlenmeyer flask cultures by inoculation. The cultures were grown at 30 °C for up to 4 days. Detailed information on cultivation can be found in [1]. A sodium dodecyl sulfate-polyacrylamide gel electrophoresis (SDS-PAGE) gel (Bio-Rad) and western blot (Bio-Rad) of the *A. niger* cultivation media after 48, 72 and 96 h containing 0, 3 or 5 copies of the *sorD* gene was made to confirm incorporation of the gene. The SDS-PAGE gel was stained with SYPRO™ Ruby protein gel stain (Invitrogen) and the western blot was visualized using TMB Enhanced One Component HRP solution (Merck).

Purification was accomplished by concentrating the cleared medium using an Amicon Ultra (Merck) concentrating filter with 50 kDa cut-off. The concentrated medium was incubated with pre-

equilibrated Ni-Sepharose resin (Cytiva) in equilibration buffer (50 mM KPi, 150 mM NaCl, pH 7) at 4 °C. The Ni-Sepharose resin was washed with 3 column volumes (CV) equilibration buffer and 3 CV wash buffer (50 mM KPi, 150 mM NaCl, 20 mM imidazole, pH 7). SorD was eluted with 1.5 CV elution buffer (50 mM KPi, 150 mM NaCl, 500 mM imidazole, pH 7). The buffer was exchanged with storage buffer (50 mM KPi, pH 7) using a PD10 desalting column or an Amicon Ultra (Merck) concentrator with 50 kDa cut-off. The concentration of SorD was determined by measuring the absorbance at 455 nm by a NanoDrop ND-100 UV/vis spectrophotometer (Thermo Scientific) and using the extinction coefficient ε_{450} = 11.3 mM^{-1} cm^{-1}. A yield of 40 mg/L of purified SorD could be obtained from 1 L of growth medium. The FAD incorporation for SorD was measured which gave an Abs_{280}/Abs_{452} ratio of around 6.0.

Cloning, Transformation and Mutagenesis of SorC. The codon-optimized synthetic gene of SorC (UniProtKB/Swiss-Prot: B6HN76.1) for *Escherichia coli* was ordered from Twist Biosciences with BsaI sites at its 5' and 3'-termini. The gene was cloned into a pBAD vector with built-in N-terminus His_6-SUMO tag with the Golden Gate methodology using the T100 Thermal Cycler (Bio-Rad).[2] For transformation, 100 ng PCR product was added to 25 µL *E. coli* NEB® 10-beta $CaCl_2$ competent cells and kept on ice for 30 min. After a 45 s heat shock at 42 °C, the cells were cooled on ice for another 5 min. Then, 500 µL LB was added for recovery and the cells were shaken at 200 rpm for 45 min at 37 °C. Thereafter, the cells were plated on LB agar plates supplemented with 50 µg mL^{-1} ampicillin and left overnight (O/N) at 37 °C. Colonies were picked and grown in 5 mL LB with 50 µg mL^{-1} ampicillin at 37 °C O/N. Subsequent plasmid isolation was done using the QIAprep® Spin Miniprep Kit (QIAGEN) and Sanger sequencing (Macrogen) was performed to confirm successful cloning. Primers for mutagenesis were ordered from Metabion and mutagenesis was performed following the QuickChange protocol (**Table S3**).[3] The PCR mix (25 µL) contained 12.5 µL PFuUltra II Hotstart PCR Master Mix (Agilent), 1 µL of 10 µM forward primer, 1 µL of 10 µM reverse primer, 1 µL of 100 ng μL^{-1} template plasmid DNA and MQ water to achieve 25 µL total volume. Mutations were confirmed by Sanger sequencing (Macrogen).

Expression and Purification of SorC. Expression was achieved by resuspending a 5 mL O/N (37 °C, 200 rpm) SorC LB culture supplemented with 50 µg mL^{-1} ampicillin into an Erlenmeyer flask containing 500 mL of terrific broth medium containing 50 µg mL^{-1} ampicillin. The flask was shaken at 200 rpm at 37 °C until an OD_{600} of 0.6 – 0.8 was reached and then induction was performed using 0.02% L-arabinose. The culture was incubated O/N at 24 °C and 200 rpm. The cells were harvested by centrifugation at 5000 rpm, 4 °C for 20 min (JA-10 rotor, Avanti JXN-26 centrifuge, Beckman Coulter) and the pellets were stored at – 20 °C. Purification was performed by resuspending cell pellets in 33% w/v lysis buffer (50 mM KPi, 150 mM NaCl, 10% v/v glycerol, pH 7), supplemented with 0.1 µM phenylmethylsulfonyl fluoride. The cells were disrupted by sonication (5 s on, 7 s off, 70% amplitude,

10 min; Branson SFX550) and the cell debris was removed by centrifugation at 10,000 rpm, 4 °C for 45 min (JA-25.15 rotor, Avanti JXN-26 centrifuge, Beckman Coulter). The supernatant was loaded on a lysis buffer-equilibrated Ni-Sepharose column (Cytiva) and was washed with 3 CV lysis buffer and 3 CV wash buffer (50 mM KPi, 150 mM NaCl, 20 mM imidazole, 10 %v/v glycerol, pH 7). Elution of SorC was achieved with 1.5 CV elution buffer (50 mM KPi, 150 mM NaCl, 500mM imidazole, 10 %v/v glycerol, pH 7). The elution buffer was exchanged with storage buffer (50 mM KPi, 10 %v/v glycerol, pH 7) using a PD10 desalting column or using an Amicon Ultra (Merck) concentrating filter with 50 kDa cut-off. Purity was confirmed by SDS-PAGE and revealed a band around 65 kDa that corresponds to the theoretical molecular weight of the 6xHis/SUMO-fused SorC. Ni-affinity purification yielded 105 mg/L of purified protein, characterized by a distinct yellow color and a typical flavoprotein absorbance spectrum with peaks at 368 nm and 452 nm. The concentration was measured with a NanoDrop ND-100 UV/vis spectrophotometer (Thermo Scientific) using the ε_{452} = 9.5 mM^{-1} cm^{-1}. The enzyme's extinction coefficient was determined to be ε_{452} = 9.5 mM^{-1} cm^{-1} following the protocol described by Macheroux et al.[4] The FAD incorporation was measured which gave an Abs_{280}/Abs_{452} ratio of approximately 11.8.

Protein Crystallization, Structure Determination, and Analysis (SorC). For crystallization screenings, the His_6-SUMO tag was cleaved off overnight using 1 mg/mL His_6-tagged SUMO protease per 100 mg/mL SorC and leaving the solution O/N at 4 °C without agitation. Thereafter, SorC was further purified using a Superdex200 100/30 Increase column (Cytiva) that was equilibrated with HEPES buffer (50 mM, pH 7.5) supplemented with 30 mM NaCl on an Äkta Pure (Cytiva) with wavelengths at 280, 360 and 450 nm. The yellow-colored fractions were pooled and concentrated to 11.2 mg mL^{-1} using Amicon Ultra (Merck) with 30 kDa cut-off. Crystallization screening was performed using the Oryx Vizier robot (Douglas Instruments) using the sitting drop method in the MCR2 96-well plate (Swissci). Dark yellow cuboid crystals were obtained in the JCSG+ screen containing 0.2 M $MgCl_2$, 0.1 M TRIS pH 8.5 and 20% PEG8000 (**Fig. S17A**). For sorbicillin-bound SorC, co-crystallized crystals were taken from the JCSG+ screen containing 0.2 M ammonium citrate dibasic and 20% PEG3350 with 11.2 mg/mL SorC, 1 mM sorbicillin and 1 mM NAD^+. Crystals were briefly soaked in a similar crystallization solution with extra 20% PEG400 before flash-cooled in liquid nitrogen. The MASSIF-1 beamline at the European Synchrotron Radiation Facility (ESRF) was used to record the X-ray diffraction data (DOI 10.15151/ESRF-ES-1542846273 and 10.15151/ESRF-ES-1552688644).[5]

Automatic data processing was done at the ESRF, using the programs autoPROC[6] for SorC and grenADES[6] for sorbicillin-bound SorC. The structure of SorC was determined by molecular replacement with Phaser[7] with an AlphaFold2 (ColabFold) model that was split in a substrate and FAD domain (due to a domain shift in the crystal structure of SorC). One monomer was in the asymmetric unit. Refinement and model building was performed with Coot[8] and REFMAC5[9]. The sorbicillin ligand was generated using the CCP4i2 aceDRG program.[10] A polder map was generated to exclude bulk solvent around

sorbicillin and FAD using Phenix.[11] The models were validated with the wwPDB Validation Service. ChimeraX was used to prepare the figures.[12] Refinement details and data collection statistics are available in **Table S1**. The experimental structure factor amplitudes and data collection statistics were deposited in the Protein Data Bank (PDB) with the following PDB IDs: 9H8M and 9H8Z.

Protein Crystallization, Structure Determination, and Analysis (SorD). For the crystallization screening, SorD was further purified using a Superdex75 100/30 column (Cytiva) that was equilibrated with HEPES buffer (50 mM, pH 7.5) supplemented with 30 mM NaCl on an Äkta Pure (Cytiva) with wavelengths at 280 and 450 nm. The yellow-colored fractions were pooled and concentrated to 9.5 mg mL^{-1} using Amicon Ultra (Merck) with 50 kDa cut-off. Crystallization screening was performed using the Oryx Vizier robot (Douglas Instruments) using the sitting drop method in the MCR2 96-well plate (Swissci). Light yellow crystals were obtained in the PEG screen containing 0.2 M potassium thiocyanate, 20% PEG3350 (**Fig. S17B**). Crystallization optimization was performed using the sitting-drop method with the 20-well Cryschem Plate (Hampton Research). For sorbicillin-bound SorD, crystals were taken from the PEG crystallization screen containing 6.0 mg/mL SorD, 1 mM sorbicillin in a crystallization solution containing 0.1 M sodium acetate pH 4.6 and 20% PEG10000. Crystals were briefly soaked in the appropriate crystallization solution with an extra 20% PEG400 before flash-cooled in liquid nitrogen. The MASSIF-1 beamline at the ESRF was used to record the X-ray diffraction data (DOI 10.15151/ESRF-ES-1656264671 and 10.15151/ESRF-ES-1552688644).[5]

Automatic data processing was done at the ESRF, using the autoPROC[6] program. The structure of SorD was determined by molecular replacement with Phaser[7] with an AlphaFold2 (ColabFold) model of SorD. One monomer of 61 kDa was in the asymmetric unit of SorD and two monomers of 61 kDa were in the asymmetric unit of sorbicillin-bound SorD. Refinement and model building was performed with Coot[8], REFMAC5[9] and Phenix[11]. The sorbicillin ligand was generated using the CCP4i2 aceDRG program.[10] The polder map was generated to exclude bulk solvent around sorbicillin and His78-linked FAD using Phenix.[11] The models were validated with the wwPDB Validation Service. ChimeraX was used to make the figures.[12] Refinement details and data collection statistics are available in **Table S1**. The experimental structure factor amplitudes and data collection statistics were deposited in the Protein Data Bank (PDB) with the following PDB IDs: 9H92 and 9H8U.

Thermostability Analyses. The thermostability analyses of SorC was determined using the *Thermo*FAD assay in duplicates.[13] In each vial, 20 µL of 20 µM SorC was prepared in buffers with different pH values. For pH values from 4.5 to 6, a citrate buffer (50 mM) was used. For pH values from 6.5 to 7.5, a KPi buffer (50 mM) was used. For pH values from 8 to 9, a TRIS buffer (50 mM) was used. Due to the temperature-dependent pKa of TRIS buffers, the pH values of the obtained melting temperatures have been shifted down by 0.5 (from pH 8 - 9 to pH 7.5 - 8.5). The T_m of SorC was also determined in KPi buffer (50 mM, pH 7) in the presence of different additives (glycerol, NaCl,

sorbicillin, NADH, NADPH, NAD^+, $NADP^+$, betaine, $MgSO_4$, $CaCl_2$, K_2SO_4, Na_2SO_4, $(NH_4)_2SO_4$, NH_4Cl, KCl, NaBr, EDTA, β-mercaptoethanol, DTT) using the same *Thermo*FAD protocol with a final SorC concentration of 20 μM. The T_m of the SorC mutants was also determined using 20 μM enzyme in KPi buffer (50 mM, pH 7) with and without addition of sorbicillin. The fluorescence was examined in a temperature range of 25 to 95 °C with a 0.5 °C min^{-1} increment.

The thermostability analyses of SorD was performed using the TychoTMNT.6 system (NanoTemper GmbH) in duplicates. In each vial, 20 μL of 20 μM SorD was prepared in buffers with different pH values. For pH values from 4.5 to 6, a citrate buffer (50 mM) was used. For pH values from 6 to 8, a KPi buffer (50 mM) was used. For pH values from 8 to 9, a TRIS buffer (50 mM) was used. Due to the temperature-dependent pKa of TRIS buffers, the pH values of the obtained melting temperatures have been shifted down by 0.5 (from pH 8 - 9 to pH 7.5 - 8.5). The T_m of SorD was also determined in KPi buffer (50 mM, pH 7) in the presence of different additives (glycerol, NaCl, sorbicillin) using the same differential scanning fluorimetry with a final SorD concentration of 20 μM. The melting curves were obtained from the intrinsic fluorescence of the tyrosine and tryptophan residues (emission at 330 nm and 350 nm) and applying a temperature gradient from 35 to 95 °C with a 30 °C min^{-1} increment. The F_{350}/F_{330} ratio was used to derive the inflection temperature (T_m).

Mass Photometry Experiments. Determination of the molecular weight and protein oligomerization of SorC and SorD were performed with the mass photometer (Refeyn, TwoMP) controlled by the AcquireMP software. A 6-well silicon gasket was fixed onto a microscope coverslip (MassGlass UC, Refeyn) and inserted into the mass photometer. A 1 μM protein stock solution was prepared in KPi buffer (50 mM, pH 7). Then, 18 μL of 0.22 μm filtered KPi buffer (50 mM, pH 7) was placed inside the well. The droplet dilution option was employed to set the focal point and, once focused, 2 μL of protein stock solution was added and mixed to reach a final protein concentration of 100 nM. Dilutions were made up to 20 nM to achieve working protein concentrations. Data collection was executed for 1 min and data processing was achieved with the DiscoverMP software (Refeyn).

Effect of pH, NaCl and Temperature on SorC. The effect of pH on the rate of oxidative dearomatization of SorC was determined using the Oxygraph+ system (Hansatech Instruments Ltd.) by measuring the oxygen depletion. The assay was performed using 16 μM sorbicillin, 200 μM NADH, 0.1 μM SorD in KPi buffers (50 mM) with different pH values (pH 6, 6.5, 7, 7.5, 8, 8.1, 8.2, 8.3 and 8.4). The measurements were performed in duplicate. The effect of NaCl on the rate of oxidative dearomatization of SorC was determined using a Cary 60 UV-Vis spectrophotometer (Agilent) by measuring substrate depletion at 400 nm. The assay was performed at 25 °C using 16 μM sorbicillin, 200 μM NADH, 0.1 μM SorD in KPi buffer (50 mM, pH 8) with varying the NaCl concentrations (0, 50, 100, 250, 500 mM). The measurements were performed in triplicate. The effect of temperature on the rate of oxidative dearomatization of SorC was also determined using a Cary 60 UV-Vis

spectrophotometer (Agilent) by measuring substrate depletion at 400 nm (**Fig. S18**). The assay was performed using 16 µM sorbicillin, 200 µM NADH, 0.1 µM SorD in KPi buffer (50 mM, pH 8) at different temperatures (15, 20, 25, 30, 35, 40, 45 and 50 °C). The measurements were performed in triplicate.

Micro-Scale Thermophoresis. The 2nd generation RED-tris-NTA dye (NanoTemper GmbH) was used for labelling SorC (mutants) and SorD following the instructions of the kit. In short, 400 nM protein diluted in KPi buffer (50 mM, pH 7) was incubated with 50 nM of dye at room temperature (RT) for 30 min. The binding assay was performed in PBS buffer supplemented with 0.05 %v/v Tween-20. The final protein concentration in the assay was 100 nM. Sorbicillin was titrated in a serial dilution and the final concentration ranged from 50 µM to 1.5 nM. A higher sorbicillin concentration range was tried for the SorC mutants and ranged from 1 mM to 30.5 nM.

pK_a Determination of Sorbicillin. The pK_a of sorbicillin was determined using the Cary 60 UV-Vis spectrophotometer (Agilent) by measuring the full spectrum from 250 to 700 nm using 20 µM sorbicillin in KPi buffer (50 mM) with different pH (5.8, 6.0, 6.3, 6.4, 6.6, 6.8, 7.0, 7.3, 7.5, 7.7, 7.8 and 8.0). These buffers were prepared by mixing different ratios of 100 mM K_2HPO_4 and 100 mM KH_2PO_4. Thereafter, the substrate samples were prepared by dissolving 0.5 µL of sorbicillin (4 mM stock in ethanol) in 99.5 µL of MQ and 100 uL of KPi buffer at different pH. The absorbance of the phenol (331 nm) and the phenolate (400 nm) were plotted against the pH. The data was fitted to a sigmoidal function and the pK_a of sorbicillin was calculated (**Fig. S11**).

Extinction Coefficient Determination of Sorbicillin. The extinction coefficient of sorbicillin was determined using a Cary 60 UV-Vis spectrophotometer (Agilent) by measuring the absorbance of different concentrations of sorbicillin (0, 5, 10, 20 and 40 µM) at 400 nm in KPi buffer (50 mM, pH 8). The concentration was plotted against the absorbance and the extinction coefficient was calculated by measuring the linear slope. The measurements were performed in duplicate.

Steady-State Kinetics. The steady-state kinetics of SorC with NAD(P)H were determined using the Cary 60 UV-Vis spectrophotometer (Agilent) by measuring the NAD(P)H depletion at 340 nm at 25 °C. The mixture contained 0.1 µM enzyme, 16 µM sorbicillin and varying NAD(P)H concentrations in KPi buffer (50 mM, pH 8). Reactions were initiated by the addition of NAD(P)H and the linear decrease of NAD(P)H (340 nm) was measured to determine the v/E values by using the molar extinction coefficient (6.22 mM^{-1} cm^{-1}). GraphPad Prism (v9.4.1 for Windows, La Jolla, CA, USA) was used to analyze the kinetic data and generate the Michaelis-Menten curve model to obtain the K_M and k_{cat} (formula: $Y = k_{cat}*X/(K_M + X)$).[14] The measurements were performed in triplicate.

The steady-state kinetics of SorC with sorbicillin were determined using a Cary 60 UV-Vis spectrophotometer (Agilent) by measuring the sorbicillin depletion at 400 nm at 25 °C. The mixture contained 0.1 µM enzyme, 200 µM NADH and varying sorbicillin concentrations in KPi buffer (50 mM, pH 8). Reactions were initiated by the addition of sorbicillin and the linear decrease of substrate (400 nm) was measured to determine the v/E values by using the molar extinction coefficient (21.86 mM^{-1} cm^{-1}). GraphPad Prism (v9.4.1 for Windows, La Jolla, CA, USA) was used to analyze the kinetic data and generate the Michaelis-Menten curve model to obtain the K_M and k_{cat} (formula: $Y = k_{cat}*X/(K_M + X)$).[14] The measurements were performed in triplicate.

The $k_{cat,app}$ of SorC with and without the addition of SorD were determined using a Cary 60 UV-Vis spectrophotometer (Agilent) by measuring the sorbicillin depletion at 400 nm at 25 °C. The mixture contained 0.1 µM SorC, 16 µM sorbicillin, 200 µM NADH in KPi buffer (50 mM, pH 8) and the optional 0.1 µM SorD. Reactions were initiated by the addition of sorbicillin and the linear decrease of substrate (400 nm) was measured to determine the v/E values by using the molar extinction coefficient (21.86 mM^{-1} cm^{-1}). The measurements were performed in triplicate.

The $k_{cat,app}$ of wild-type and mutant SorC enzymes was measured using three techniques: measuring NADH depletion at 340 nm, measuring sorbicillin depletion at 400 nm, and measuring oxygen depletion at 25 °C. All experiments contained 0.1 µM enzyme, 16 µM sorbicillin, 200 µM NADH in KPi buffer (50 mM, pH 8) at 25 °C. The UV-Vis experiments were performed using a Cary 60 spectrophotometer (Agilent) and the oxygen depletion experiments were performed using the Oxygraph+ (Hansatech Instruments Ltd.) All the measurements were performed in duplicate.

NAD(P)H oxidation of the wild-type and mutant SorC enzymes was determined using a Cary 60 UV-Vis spectrophotometer (Agilent) by measuring the NAD(P)H depletion at 340 nm at 25 °C. The mixture contained 0.1 µM enzyme and 200 µM NADH in KPi buffer (50 mM, pH 8). Reactions were initiated by the addition of NAD(P)H and the linear decrease of NAD(P)H (340 nm) was measured to determine the v/E values by using the molar extinction coefficient (6.22 mM^{-1} cm^{-1}). The percentage of NAD(P)H oxidation was calculated by dividing it through the $k_{cat,app}$ at 340 nm in the presence of 16 µM sorbicillin.

Liquid Chromatography – Mass Spectroscopy Experiments. For the *in vitro* biochemical assay of SorC and SorC E245 mutants, 2 µM of enzyme was incubated with 2 mM sorbicillin (50 mM stock in acetone; **Fig. S20-S21**) and 4 mM NADH in 100 µL KPi buffer (50 mM, pH 8) at 30 °C and 200 rpm for 60 min. The reaction was quenched with 1900 µL MeOH and subsequently centrifuged at 15,000 rpm for 5 min before UHPLC/HRMS analysis. Control reactions were run and included only sorbicillin, SorC with only sorbicillin or SorC with only NADH.

For the *in vitro* biochemical assay of SorC with SorD, 10 µM of SorC and 10 µM SorD were incubated with 2 mM sorbicillin (50 mM stock in acetone) and 4 mM NADH in 100 µL KPi buffer (50 mM, pH 8) at 30 °C for 20 h. The reaction was quenched with 900 µL ACN and subsequently centrifuged

at 15,000 rpm for 5 min UHPLC/HRMS analysis. Control reactions were run and included only sorbicillin and SorD with only sorbicillin.

The ExionLC TM AD LC unit was used for all analyses and contained a column oven set at 40 °C, an autosampler set at 10 °C and a binary gradient pump. The MS device was a high resolution QTOF mass spectrometer (AB Sciex X500B) with a Turbo V Ion source and Twin Sprayer electrospray ionization (ESI) probe. The software SCIEX OS 2.1 was used for analyses of the samples. The injection volume was 10 µL. The machine was equipped with a Kinetex EVO C18 100 Å column (100 mm length X 2.1 mm diameter, 2.6 µm particle size; Phenomenex). The mobile phase consisted of (A) water and (B) acetonitrile both supplemented with 0.1 %v/v formic acid. The flow rate was set at 0.3 mL min^{-1}. For the SorC and SorC E245 mutant reactions the gradient was the following: 5% B at 0.0-1.0 min, 5-95% B at 1.0-16.0 min, 95% B at 16.0-24.0 min, 95-5% B at 24.1-26.0 min. For the combined SorC and SorD reactions, the gradient was the following: 2% B at 0.0-1.0 min, 2-95% B at 1.0-16.0 min, 95% B at 16.0-18.0 min, 95-2% B at 18.1-20.0 min. For the mass spectra, a positive polarity was used with the following instrument parameters: 30 psi curtain gas, 450 °C temperature, 50 V declustering potential, 45 psi ion source gas 1, 55 psi ion source gas 2, 5500 V ion spray voltage, 10 V collision energy. The biotoolKit extension of SciexOS 2.1 was employed for mass deconvolution using a m/z input range of 50-1000 Da.

2. Supplementary tables

Table S1. Crystallographic data collection and refinement statistics. Sorbicillin is named A1ITD.

	**SorC**	**SorC: A1ITD**	**SorD**	**SorD: A1ITD**
PDB entry	9H8M	9H8Z	9H92	9H8U
Wavelength (Å)	0.96546	0.96546	0.96546	0.96546
Resolution range (Å)	67.45 – 1.38 (1.40 – 1.38)	42.32 – 1.71 (1.74 – 1.71)	137.43 – 1.55 (1.58 – 1.55)	290.43 – 3.0 (3.18 – 3.00)
Space group	$P2_12_12_1$	$P2_12_12_1$	$C222_1$	$P4_32_12$
Unit cell (Å)	45.81292.83 98.16	42.32 90.96 94.55	65.11 115.72 137.43	90.57 90.57 290.43
Total reflections	379985 (14715)	165546 (7416)	494466 (21068)	247786 (15607)
Unique reflections	85417 (4104)	38849 (1814)	75455 (3698)	24425 (3287)
Multiplicity	4.4 (3.6)	4.3 (4.1)	6.6 (5.7)	10.1 (4.7)
Completeness (%)	98.8 (97.0)	96.4 (86.8)	100.0 (100.0)	96.8 (83.4)
Mean I/sigma(I)	13.4 (1.6)	13.1 (1.5)	6.9 (1.3)	10.0 (1.4)
$CC_{1/2}$	0.999 (0.543)	0.999 (0.528)	0.995 (0.578)	0.995 (0.467)
R_{merge}	0.043 (1.028)	0.048 (0.876)	0.157 (1.381)	0.160 (1.157)
R_{work}/R_{free}	0.178 (0.317)/0.202 (0.321)	0.172 (0.316)/0.227 (0.321)	0.169 (0.309)/0.202 (0.329)	0.206 (0.359)/0.286 (0.437)
No. of atoms	3760	3547	4323	7514
- protein	3267	3221	3529	7002
- ligands	67	67	190	503
- water	426	259	604	9
RMS bond length (Å)	0.010	0.0074	0.0086	0.0118
RMS bond angles (°)	1.889	1.618	1.750	1.329
Ramachandran favored (%)	98.0	97.0	98.0	87.6
Ramachandran allowed (%)	2.0	3.0	2.0	1.5
Ramachandran outliers (%)	0.0	0.0	0.0	0.14
Average B-factor ($Å^2$)	24.0	30.0	19.0	77.0
- protein	23.2	29.4	17.2	76.3
- ligands	25.0	33.7	30.8	96.6
- water	33.4	37.9	30.3	65.0

In parentheses the statistics for the highest-resolution shell are given.

Table S2. Activity data displayed in Figure 4.

Mutant	Substrate depletion (s^{-1})	NADH depletion (s^{-1})	Oxygen depletion (s^{-1})	NADH depletion without substrate (s^{-1})	Uncoupling (%)[a]
WT	3,30	3,63	3,59	0,05	8,08
E245A	0,01	0,15	0,21	0,15	93,09
E245D	0,01	0,21	0,31	0,09	95,85
E245Q	0,01	0,10	0,31	0,07	97,08
R201A	0,14	0,28	0,25	0,03	44,42
W300A	0,12	0,26	0,28	0,15	55,85
T51A	0,61	1,24	1,02	0,11	39,85
Y114A	0,73	1,02	1,25	0,06	41,35
H243A	1,53	2,60	1,55	0,04	1,48
W419A	1,20	1,82	2,09	0,04	42,46

[a] Uncoupling measures the wasteful conversion of oxygen to hydrogen peroxide or superoxide. It is calculated as (1-substrate depletion/oxygen depletion) x 100.

Table S3. Primers used in this study.

Primer	Sequence (5'-3')
Pc_sorD_P1f	CCAGCATCATTACACCTCAGCAATGCAGGCCGCCAGTGCATTTG
Pc_sorD_P2r	ATTGATTGTCTAGTGATGGTGATGGTGATGGGACAGAGGTGGGATACTCTG
SorC_T51A_FW	TGGGTGTTGGGATAGCGTTCAC
SorC_T51A_RV	CATTGGGAGTGAACGCTATCCCAAC
SorC_Y114A_FW	CGTAAAAGGTGGCGCCAAGG
SorC_Y114A_RV	CAGGCCTTGGCGCCACC
SorC_R201A_FW	CAACTTGGCTACGCGGGTATGG
SorC_R201A_RV	GGAACCATACCCGCGTAGCC
SorC_E245A_FW	GCCATGCATATAGCGGCATTTATTATGGAC
SorC_E245A_RV	CCTTGTCCATAATAAATGCCGCTATATGCAT
SorC_E245D_FW	CATGCCATGCATATAGATGCATTTATTATGG
SorC_E245D_RV	TCCTTGTCCATAATAAATGCATCTATATGC
SorC_E245Q_FW	CATGCCATGCATATACAGGCATTTATTATGG
SorC_E245Q_RV	TCCTTGTCCATAATAAATGCCTGTATATGC
SorC_H243A_FW	GAAGTACATGCCATGGCGATAGAAGC
SorC_H243A_RV	CATAATAAATGCTTCTATCGCCATGGCATG
SorC_W300A_FW	GAAAAGCTTGAGAAGGCGGCGGTG
SorC_W300A_RV	GTCGAACACCGCCGCCTTCTC
SorC_W419A_FW	GTCACCAACTGGCGGACCATG
SorC_W419A_RV	CAGTGTCATGGTCCGCCAGTTG

Table S4. Fasta of native SorC and SorD. SorC from *Penicillium chrysogenum* (UniprotKB/Swiss-prot: B6HN76.1) and SorD from *Penicillium chrysogenum* ATCC 48271.

Protein	Fasta
SorC	MTRSANSPFEVAIVGGGITGLALAVGLLKRNVSFTIYERAENFGELGVGITFTPNAQRA<br>MEALDPCVLQSFTNVASAPSGGTINFVDGVREQGSEDPRTSTAALLFQLHVKGGYKAC<br>RRCDFVDQIVQHIPKDCVQYRKWLDSIETDHESGRAVLKFRDGEIAHADVVIGCDGIRS<br>QVRASMFGTDELCPRAQYSHQLGYRGMVPLAQATAVLGPEKTSSAVLHTGPGAFVLTI<br>PLAEVHAMHIEAFIMDKEEWPEVQTSSDSKRYVLPATRNEATKAFAEFGPTVRSAVSM<br>FPEKLEKWAVFDMLEAPVPTFAKGRVCLAGDAAHASTPNQGGGAGFGIEDALVLAEV<br>LAVLAEAPNVSGIVASEALAVYSEVRYERSQWLVRSSRRTGELCTWKDRDWGLAAEE<br>LSRDIISRSHQLWDHDTAGMVSDALAILGERVRGADTAF
SorD	MQAASAFATCLLASVGGNSSAVAFPNQANYSTLVAPYNFDLLTTPSAIVWPQDTQQVAA<br>AVKCAVDSDIKVQPKSGGHNYGNYGSTTGELSVNLDNLQHFSMNETSWTARLGPGNR<br>LGRVTELMYNNGGRHVPHGTTFTVGLGGHATVGGAGAASRMHGLLLDYVEEVEVVL<br>ANSSIVRASKSHNEDLFFAVRGAASSVGIVTDFSIRTEPVPVSSVTYSYIWEGTDPAARAE<br>VFLTWQSLLAGGSLPQHMAYDLVATANSMILGGAYFGSQEDFEAFNLSSHFKVAPDVTH<br>IKTYTNFFDFSAAASAQTKAAGIASPSHFYAKSLVFNQQTLIPDDAAEEVFKYLATTKNG<br>TDLYAVTFAALGGAVRDVSASETAFYHRDASYFMFSFGRTSGDLTDTTVQFLDGLSEVL<br>TSGQPDAYYGQYVGNVDPRQPTDEALTGYYGKNLHRLQQIKSAVDPNDVFHNQQSIPP<br>LS

3. Supplementary figures

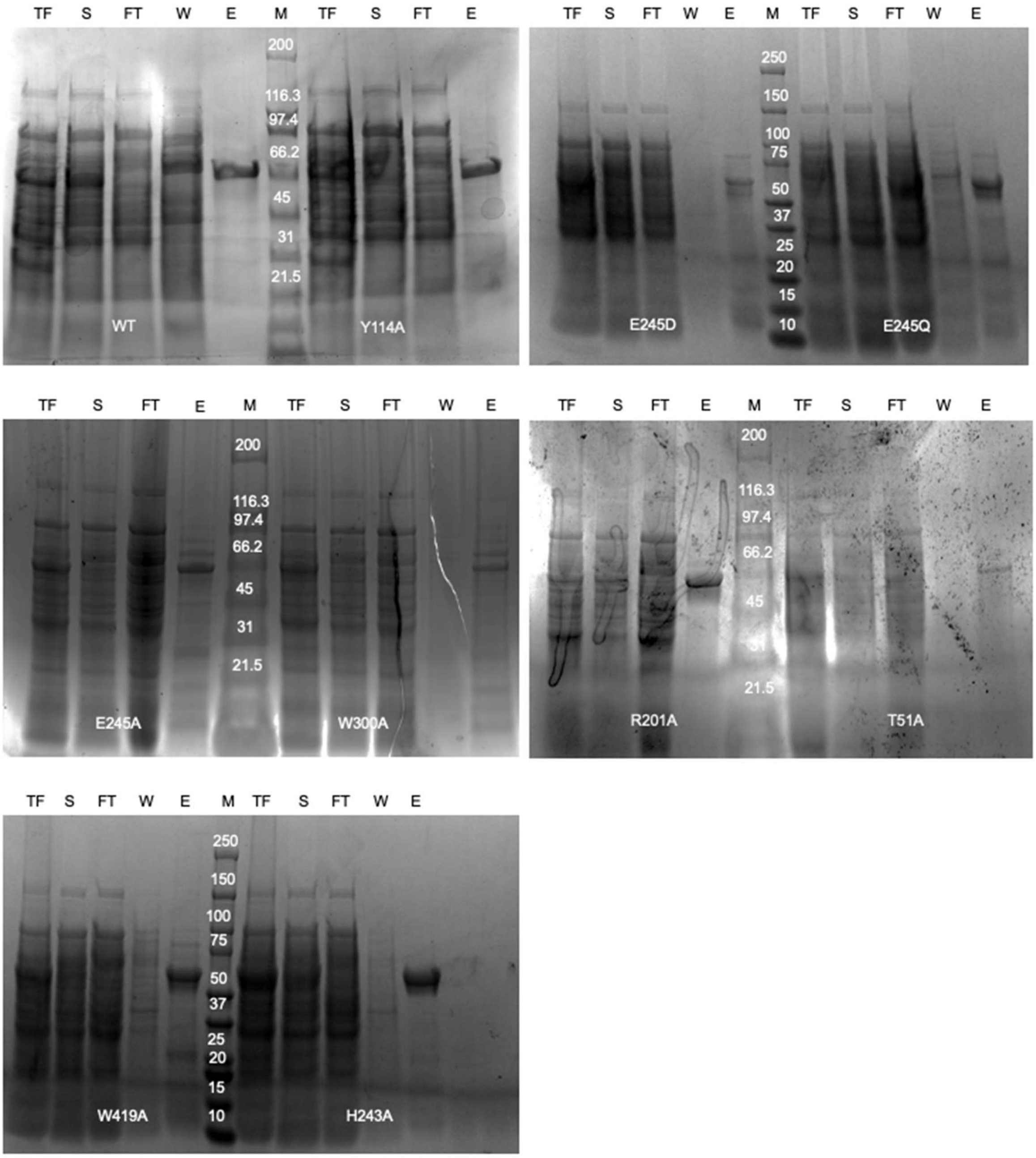

Figure S1. Uncropped SDS-PAGE gels of purified His$_6$-SUMO-tagged SorC wild-type (WT) and mutants. (TF: total fraction, S: supernatant, FT: flow-through, W: wash, E: elution). The gels were stained with Coomassie blue R-250.

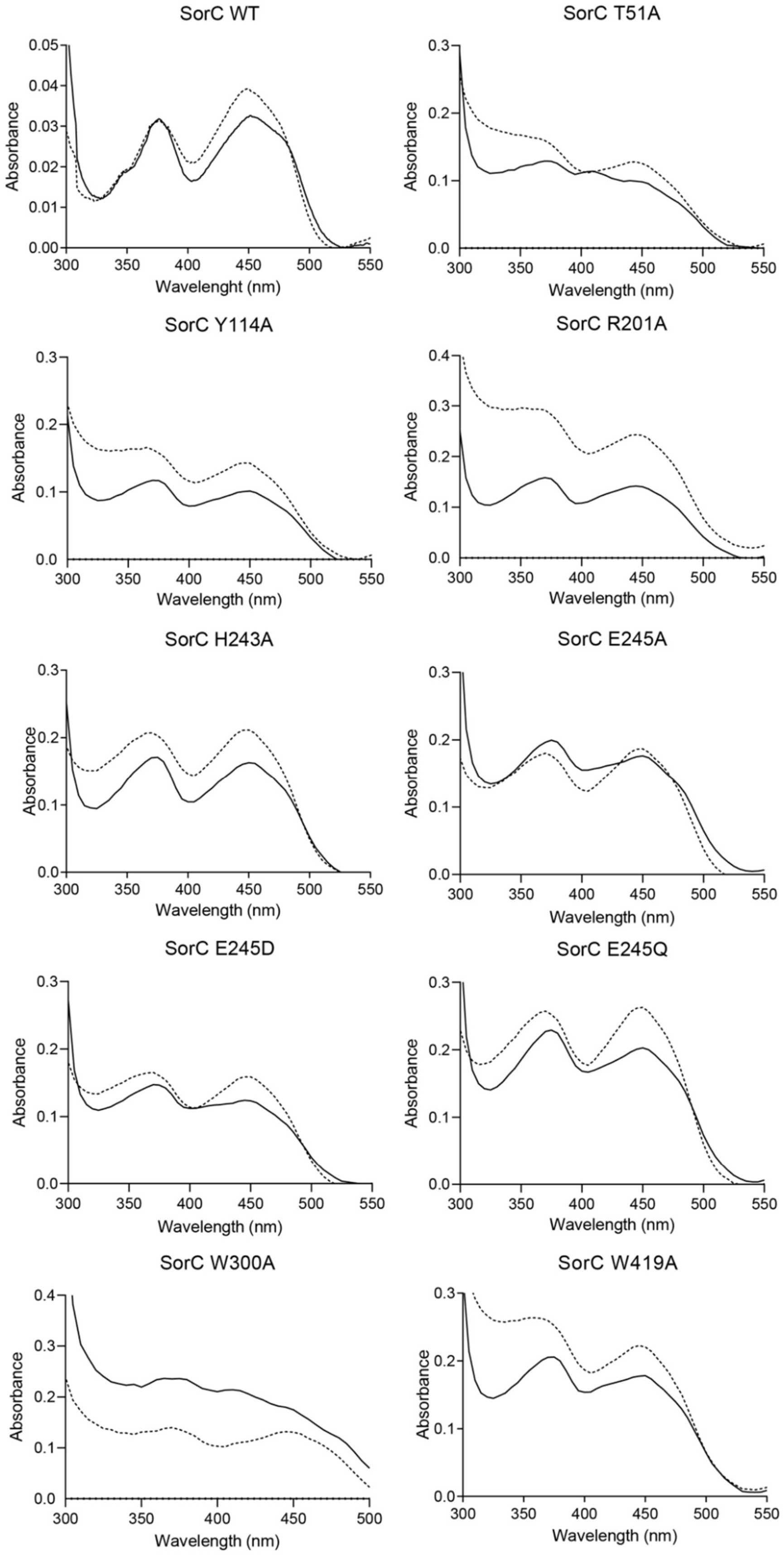

Figure S2. UV-vis absorbance spectrum of purified SorC (20 μM) wild-type and mutants in native (solid lines) and denatured states (dotted lines).

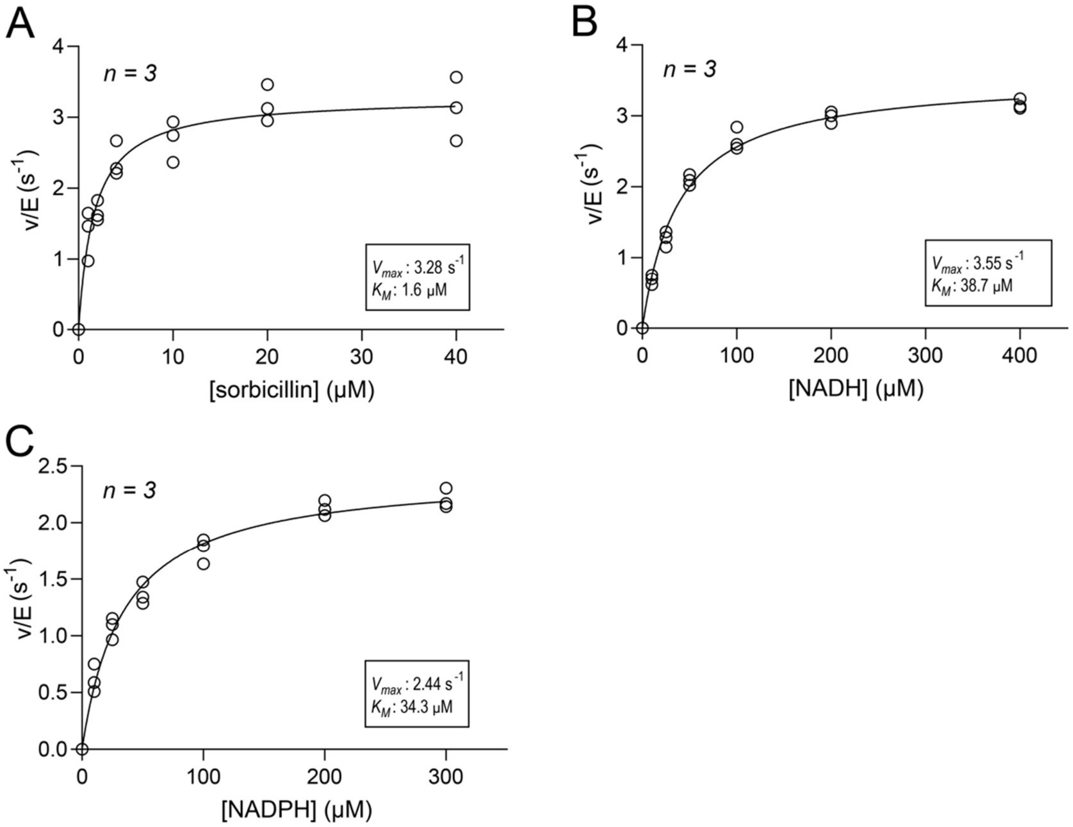

Figure S3. Dependence of initial rates on substrate concentrations for wild-type SorC (0.1 μM). (A) Varying sorbicillin concentrations using 200 μM NADH. **(B)** Varying NADH concentrations using 16 μM sorbicillin. **(C)** Varying NADPH concentrations using 16 μM sorbicillin.

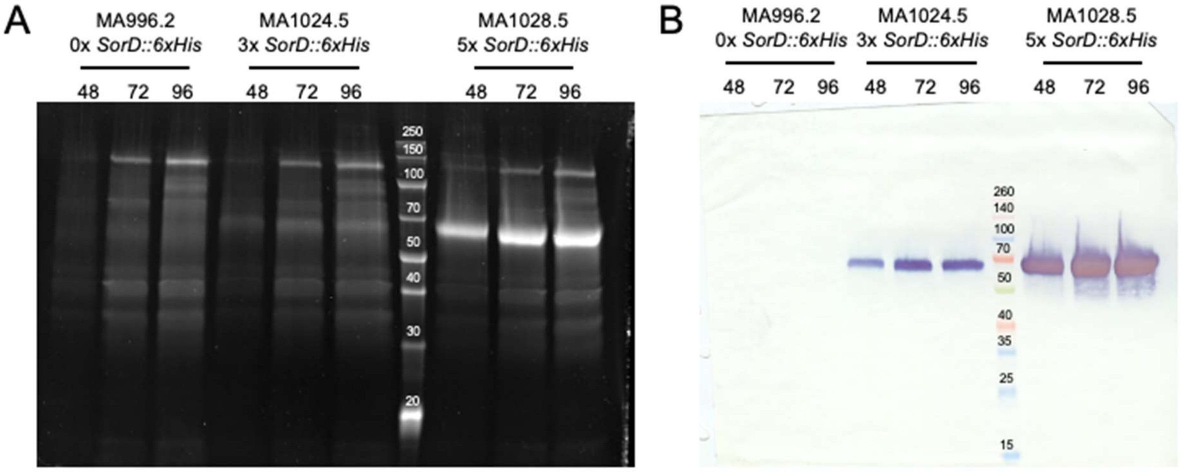

Figure S4. Different number of insertions of *sorD* into the genome of *Aspergillus niger*. (A) SDS-PAGE gel of the *A. niger* cultivation media after 48, 72 and 96 h containing 0, 3 or 5 copies of the *sorD* gene. The gel was stained with Invitrogen SYPRO^{TM} Ruby protein gel stain. **(B)** Anti-His Western blot of the *A. niger* cultivation media after 48, 72 and 96 h containing 0, 3 or 5 copies of the *sorD* gene. The blot was visualized using Sigma TMB Enhanced One Component HRP solution.

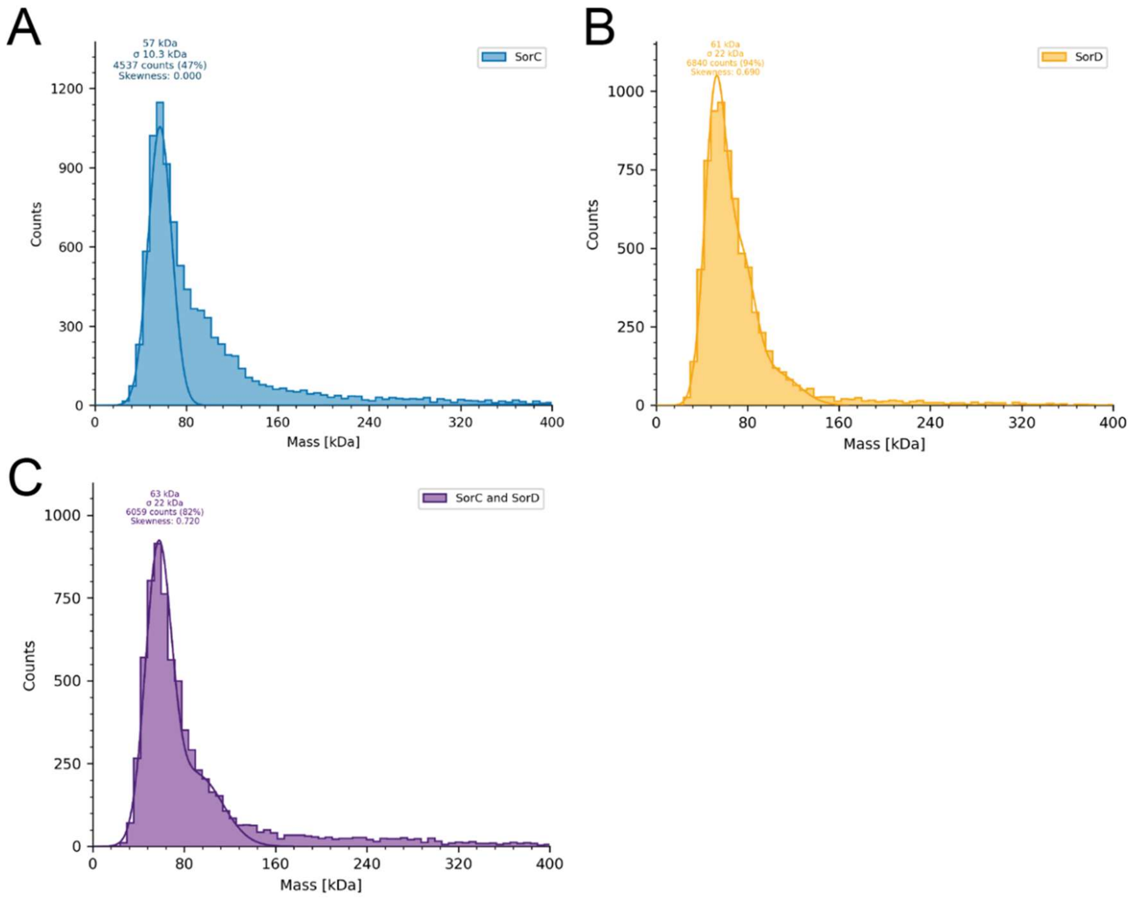

Figure S5. Mass photometry analysis. The mass distribution of SUMO-tagged SorC **(A)**, glycosylated SorD **(B)** and SUMO-tagged SorC combined with glycosylated SorD **(C)**. The protein concentrations are 20 nM**.**

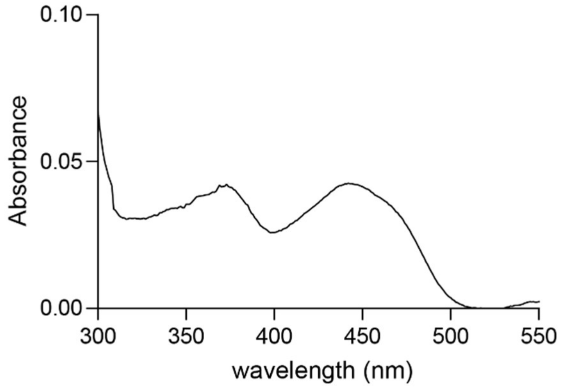

Figure S6. UV-vis absorbance spectrum of purified SorD (20 μM).

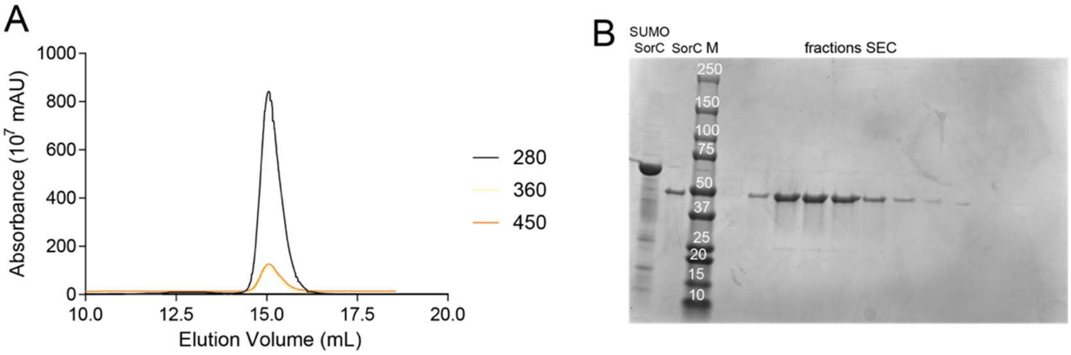

Figure S7. Size-exclusion chromatography of SorC. (A) The chromatogram of SUMO-cut SorC using the Superdex200 100/30 increase column. **(B)** Uncropped SDS-PAGE gel of SorC before and after addition of SUMO protease, and different fractions after size-exclusion chromatography. The gel was stained with Coomassie blue R-250.

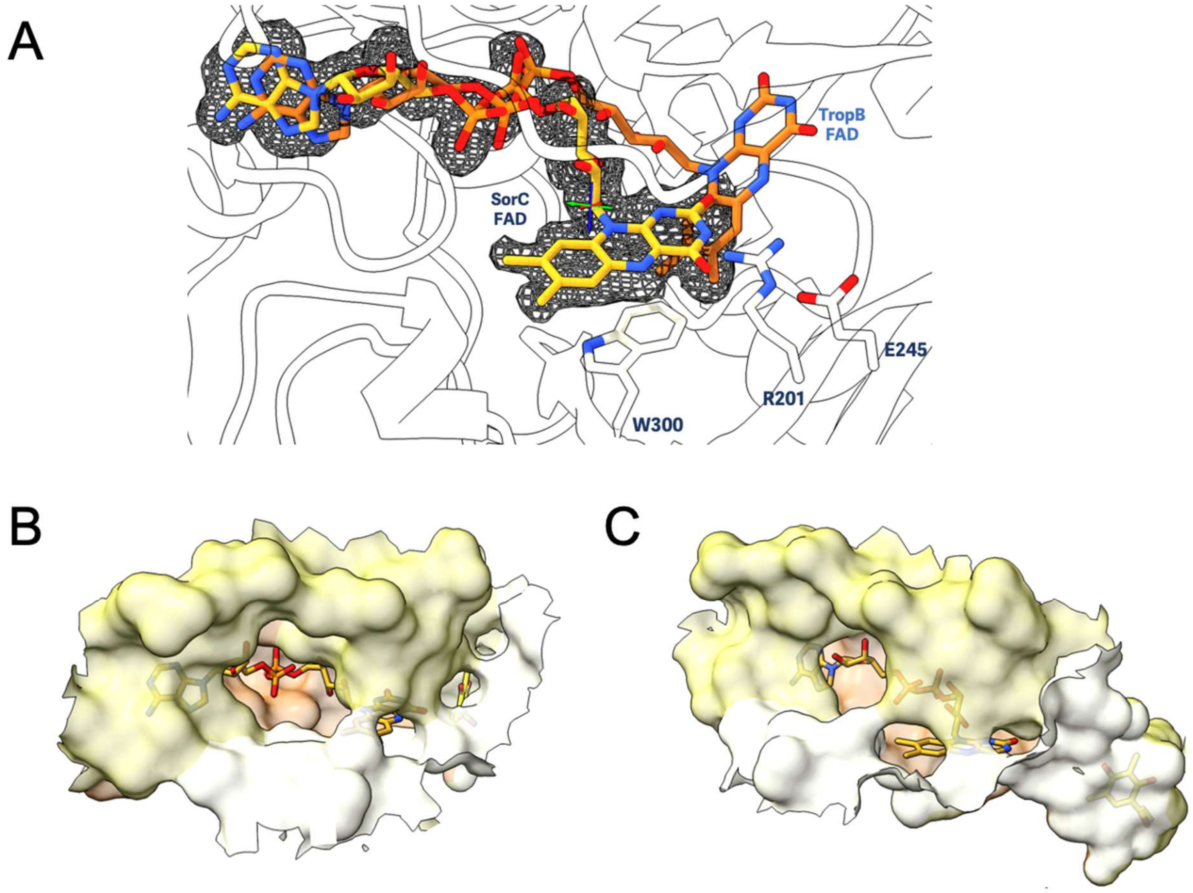

Figure S8. The flavin positioning in SorC. (A) The polder omit map for the FAD contoured at 1 σ level demonstrates that FAD (light orange) is in the *out* conformation. The predicted *in* conformation of FAD (dark orange) is shown for reference (**Fig. 3D**). **(B)** The opening near to the monophosphate moiety of FAD. **(C)** The opening near to the isoalloxazine ring of FAD and the opening towards the substrate pocket with sorbicillin bound.

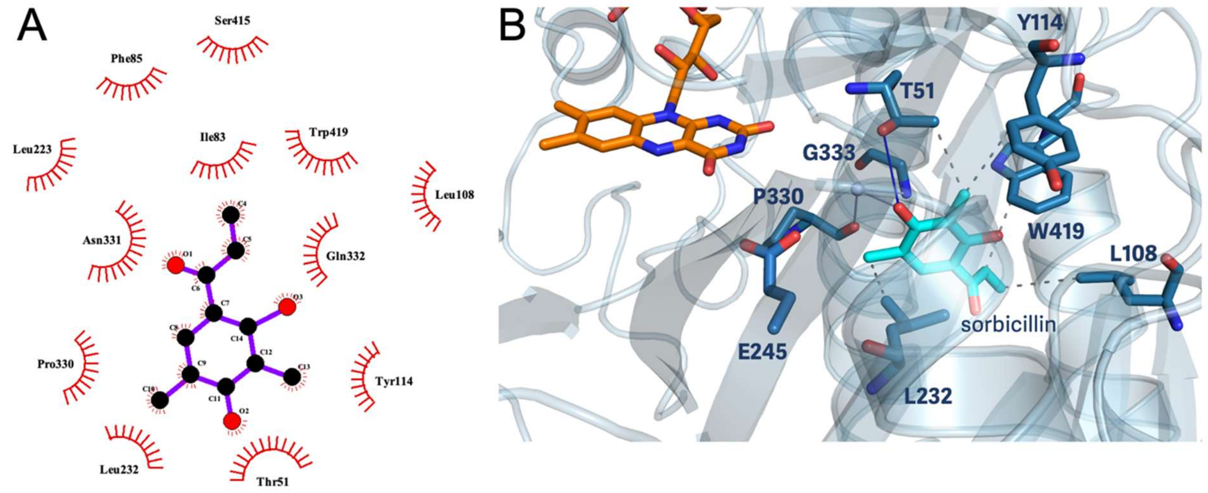

Figure S9. The interactions between SorC and sorbicillin. (A) A LigPlot-style representation of the hydrophobic binding interactions of sorbicillin with the residues shown. **(B)** Binding interactions by Protein-Ligand Interaction Profiler (PLIP) with hydrogen-bonding interactions in blue lines, hydrophobic interactions in dashed grey lines and water bridges in grey lines. The grey dot is a water molecule.

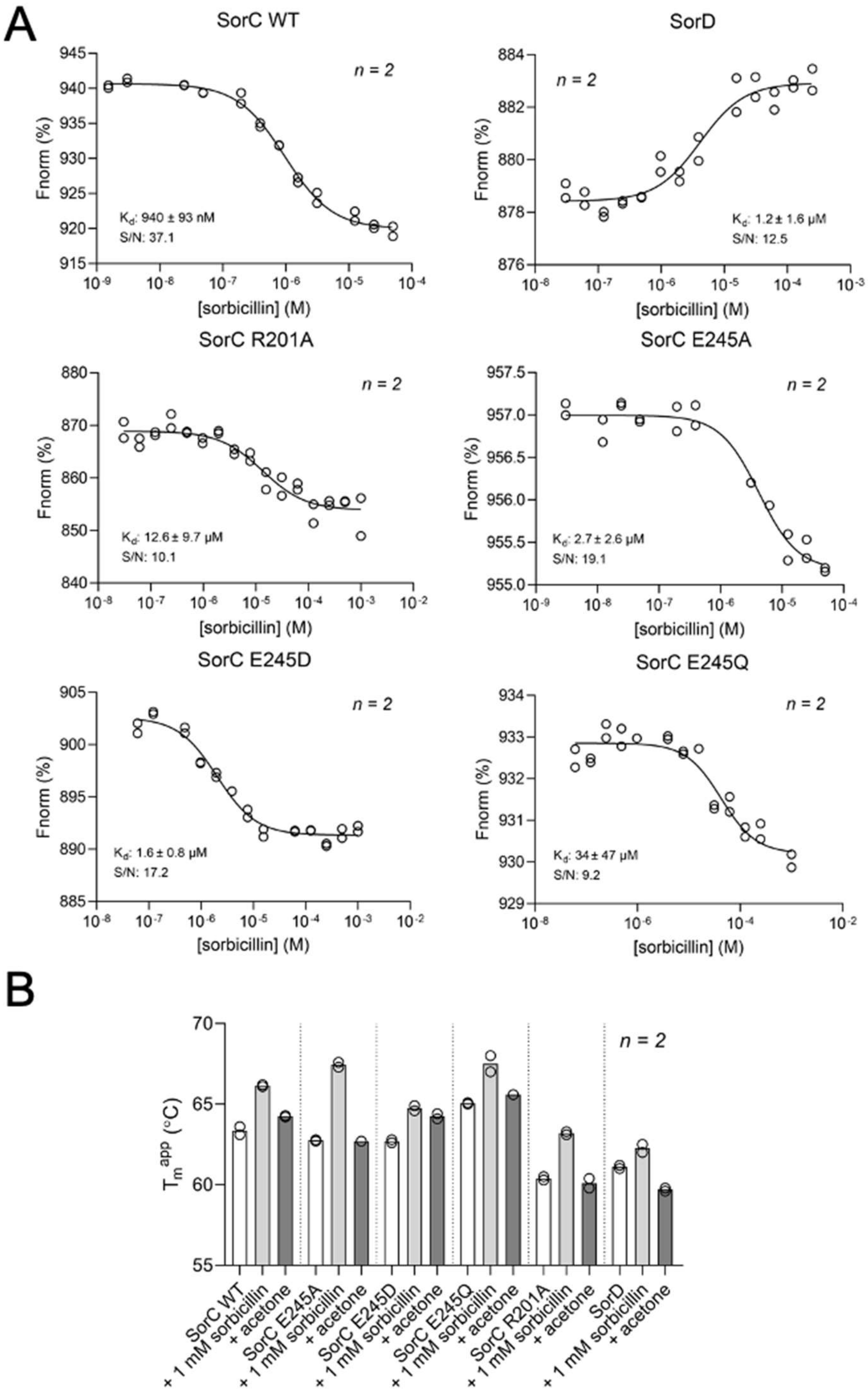

Figure S10. Substrate binding to SorC and SorD. (**A**) The dissociation curves of sorbicillin with wild-type SorC, SorC mutants, and SorD. The analysis was performed using microscale thermophoresis. (**B**) Label-free measurements of thermal shifts induced by sorbicillin binding to SorC and SorD proteins using Tycho NT.6.

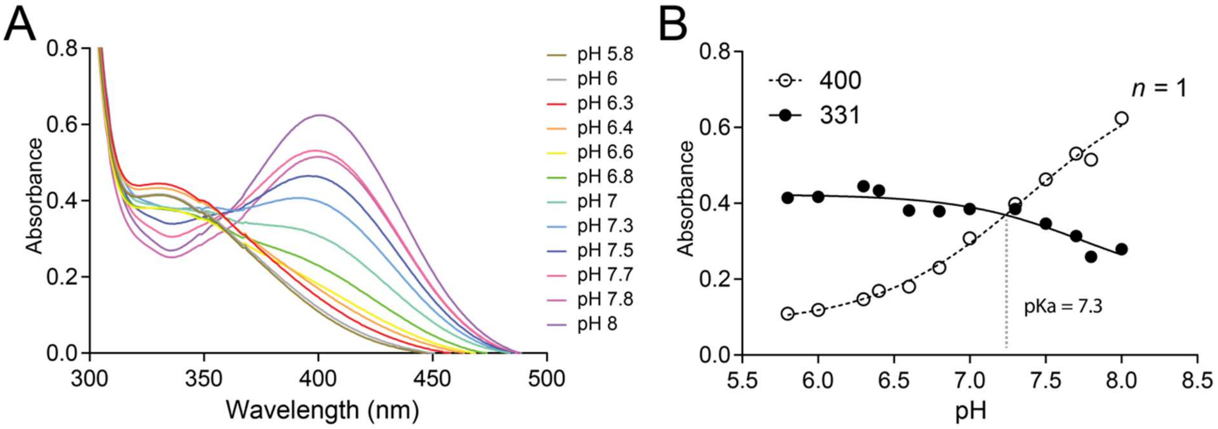

Figure S11. pKa determination of sorbicillin. (A) The full UV-vis absorbance spectrum of 20 µM sorbicillin at different pH values. **(B)** The difference in absorbance at 331 nm (protonated form) and 400 nm (phenolate form). The pK_a lies at the intersect at a pH of 7.3.

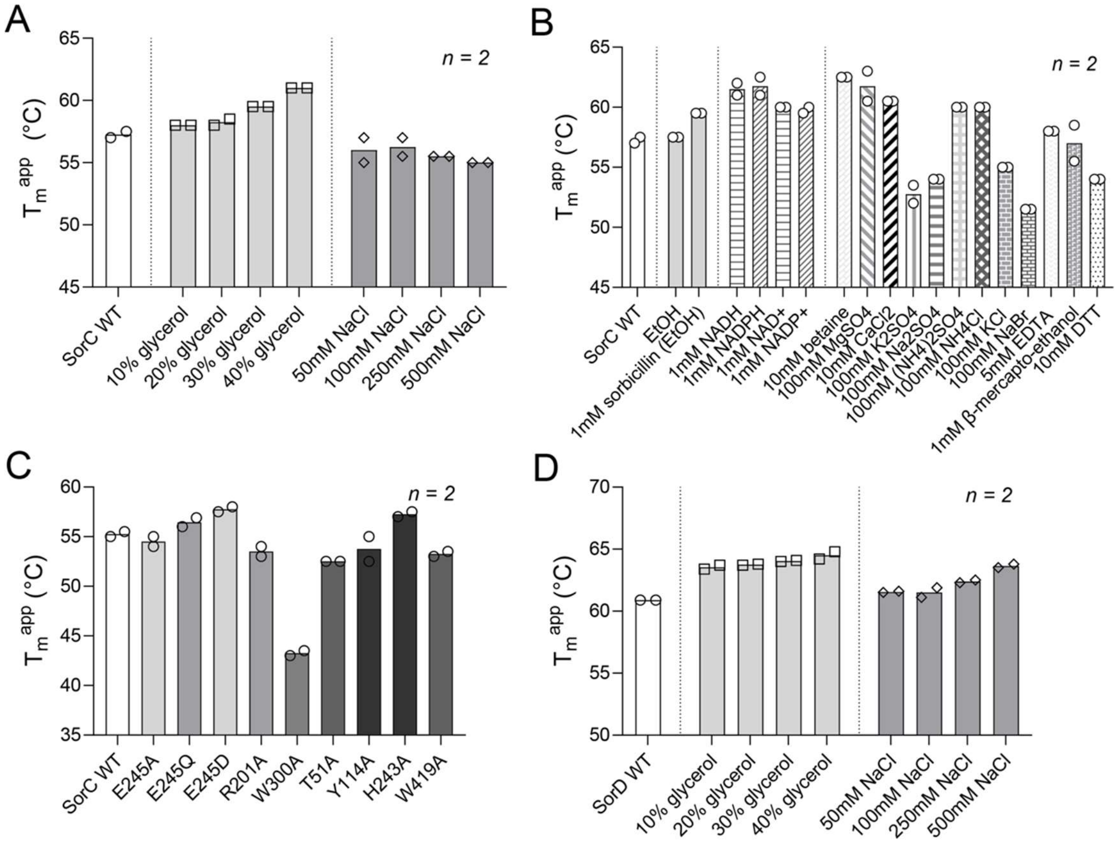

Figure S12. The melting temperatures of wild-type SorC, SorC mutants and SorD. (A) The effect of glycerol and NaCl on the melting temperature of wild-type SorC. **(B)** The effect of different additives on the melting temperature of wild-type SorC. **(C)** The comparison of wild-type SorC with the different SorC mutants. **(D)** The effect of glycerol and NaCl on the melting temperature of SorD. All controls were performed in 50 mM KPi pH 7.

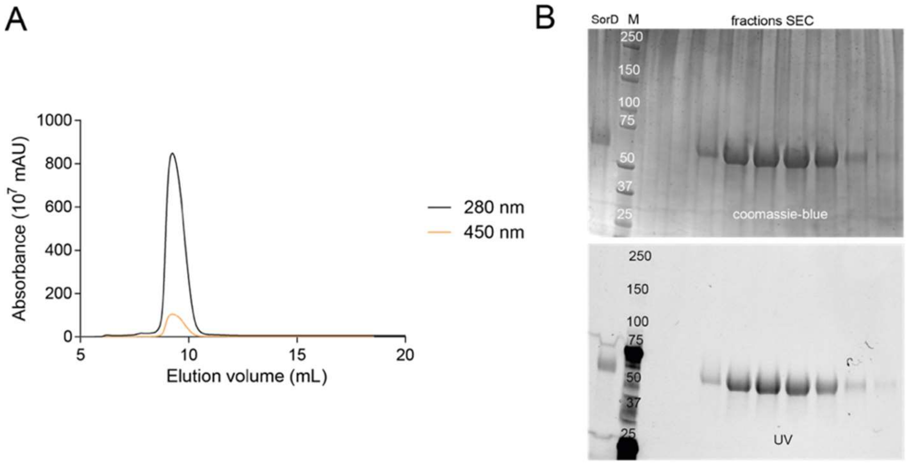

Figure S13. Size-exclusion chromatography of SorD. (A) The chromatogram of glycosylated SorD using a Superdex75 100/30 column. **(B)** Uncropped SDS-PAGE gel of SorD before and after size-exclusion chromatography with different elution fractions. The top gel was stained with Coomassie Blue R-250. The bottom gel was stained with acetic acid and visualized by UV-illuminated light of the covalently-bound FAD to His78 of SorD.

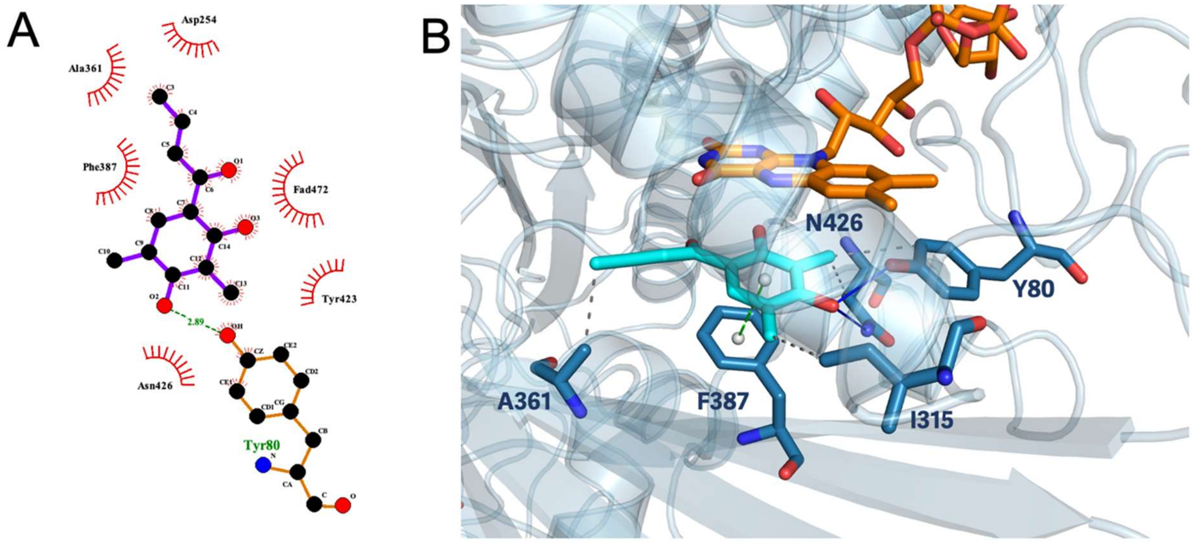

Figure S14. The interactions between SorD and sorbicillin. (A) A LigPlot-style representation of the binding interactions of sorbicillin with the residues shown. **(B)** Binding interactions by Protein-Ligand Interaction Profiler (PLIP) with hydrogen-bonding in blue lines, hydrophobic interactions in dashed grey lines and by $\pi - \pi$ stacking interactions in dashed green lines with grey dots.

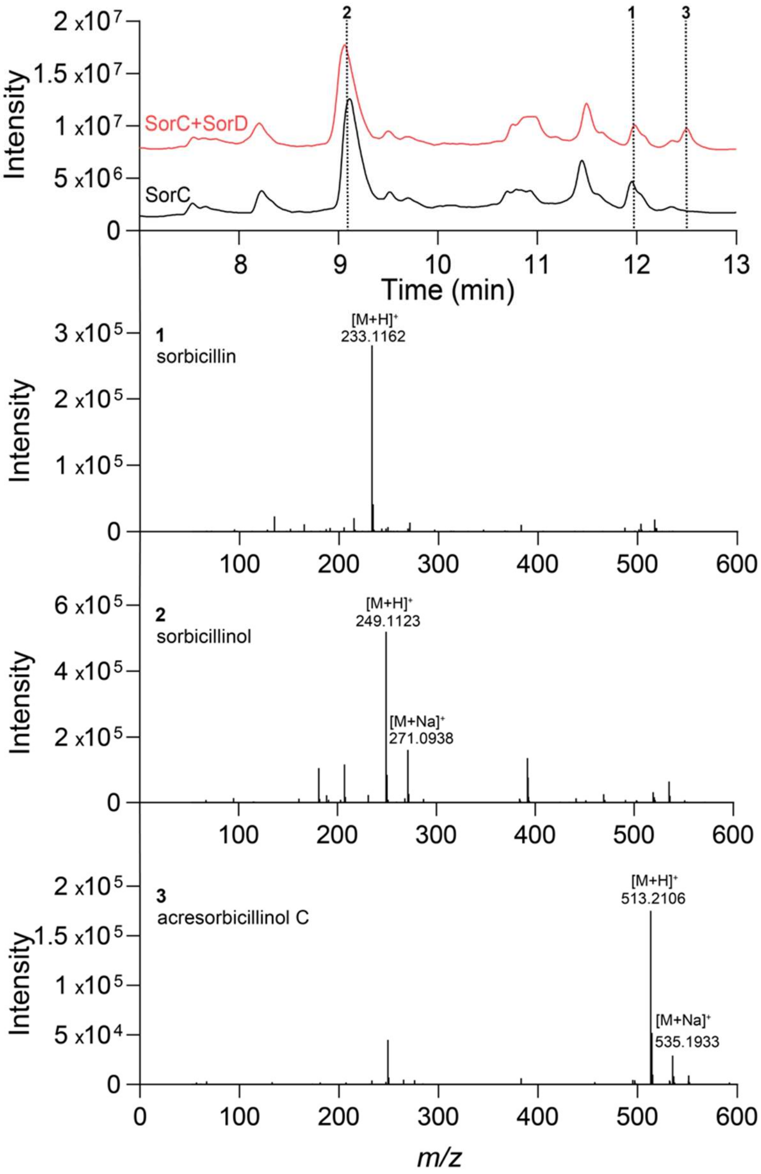

Figure S15. The LC-MS chromatogram of SorC with and without SorD, including the mass spectra related to the substrate and different products. The reaction contained 10 µM SorC with or without 10 µM SorD, 2 mM sorbicillin, 4 mM NADH in 50 mM KPi pH 8 at 30 ºC and was run for 20 h.

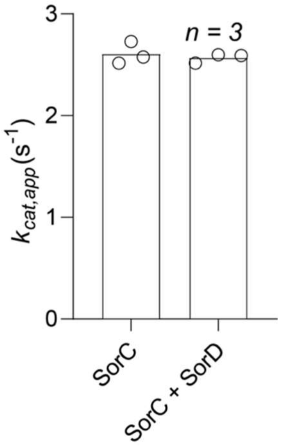

Figure S16. The difference in sorbicillin conversion between SorC with and without the addition of SorD. The rate was obtained by measuring substrate depletion at 400 nm. Reaction mixtures contained 0.1 µM of enzyme, 16 µM sorbicillin, 200 µM NADH in 50 mM KPi pH 8 at 25 °C.

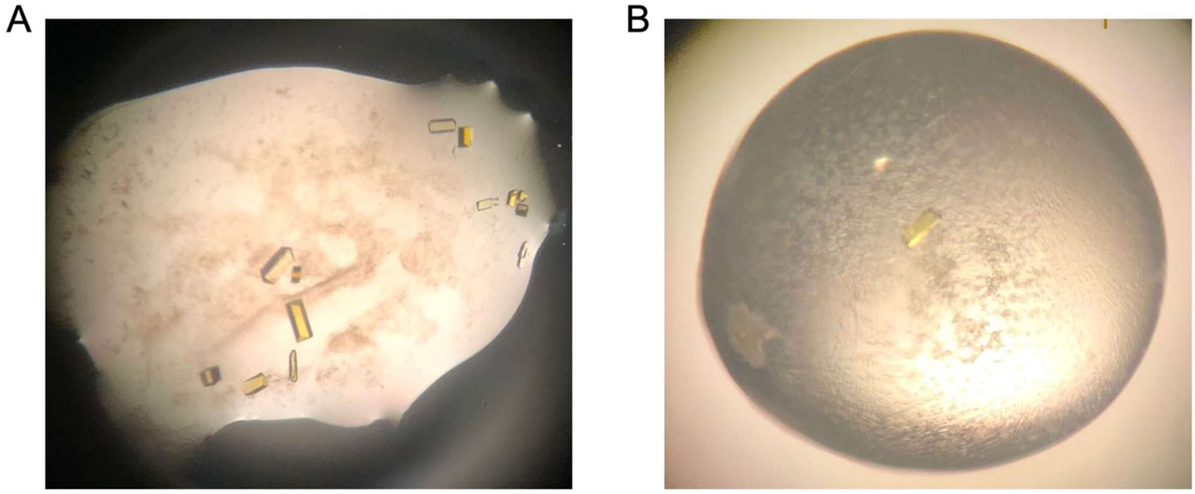

Figure S17. Crystals of SorC and SorD. (A) Dark yellow cuboid crystals of SorC in the JCSG+ D6 screening condition. **(B)** Lighter yellow crystal of SorD in the PEG G4 screening condition.

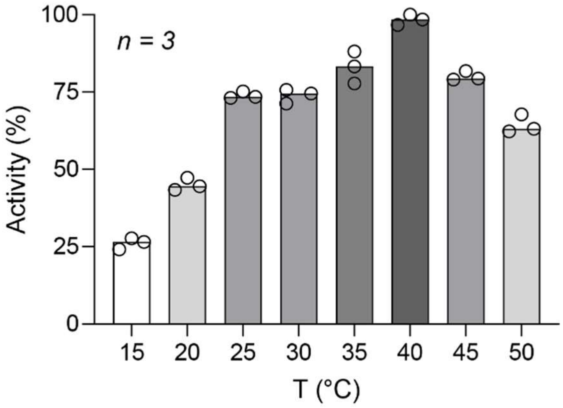

Figure S18. The activity of wild-type SorC toward sorbicillin at different temperatures. The reactions contained 0.1 µM SorC, 16 µM sorbicillin, 200 µM NADH in 50 mM KPi pH 8.

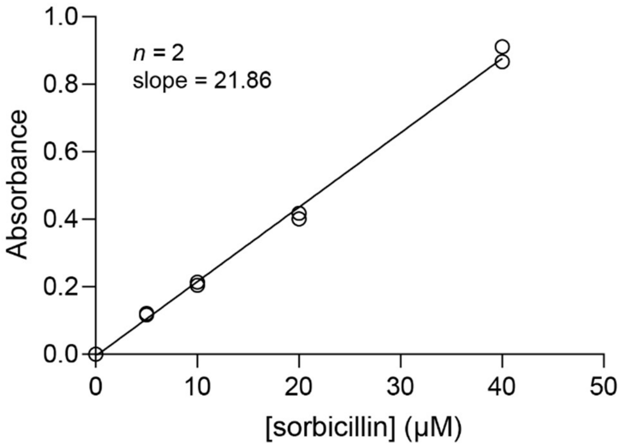

Figure S19. The extinction coefficient at 400 nm of sorbicillin calculated by measuring the slope of the absorbance of different sorbicillin concentrations.

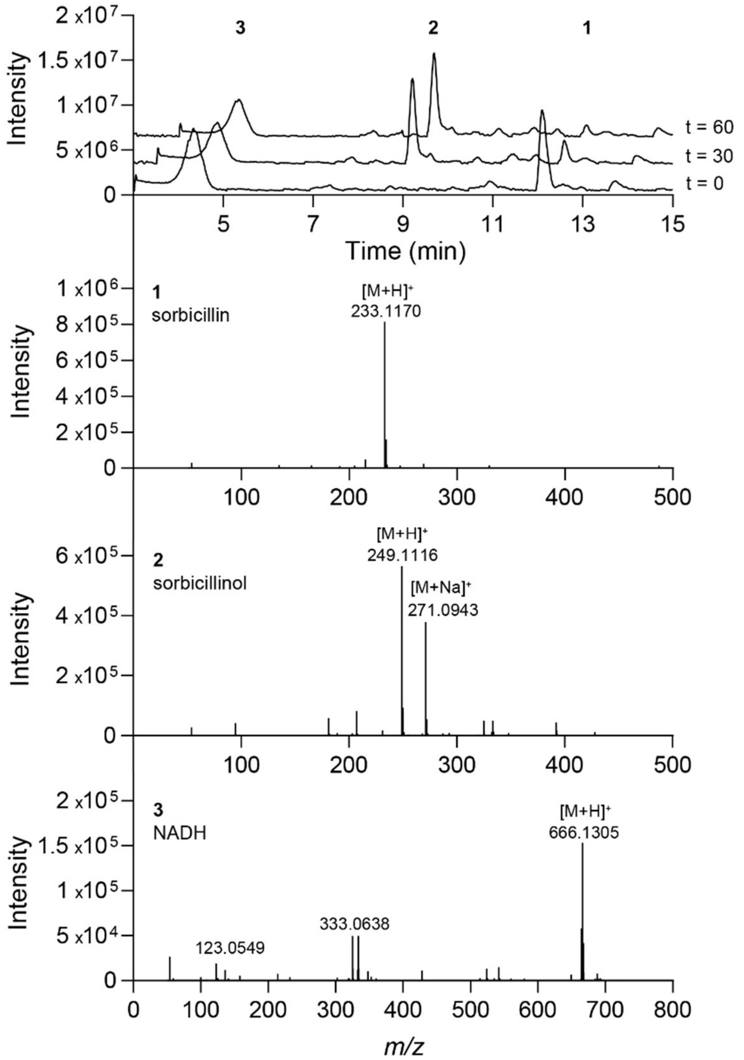

Figure S20. The LC-MS chromatogram of sorbicillin conversion by SorC over time, including the mass spectra related to the different peaks. The reaction contained 2 μM SorC, 2 mM sorbicillin, 4 mM NADH in 50 mM KPi pH 8 at 30 °C for 1 h.

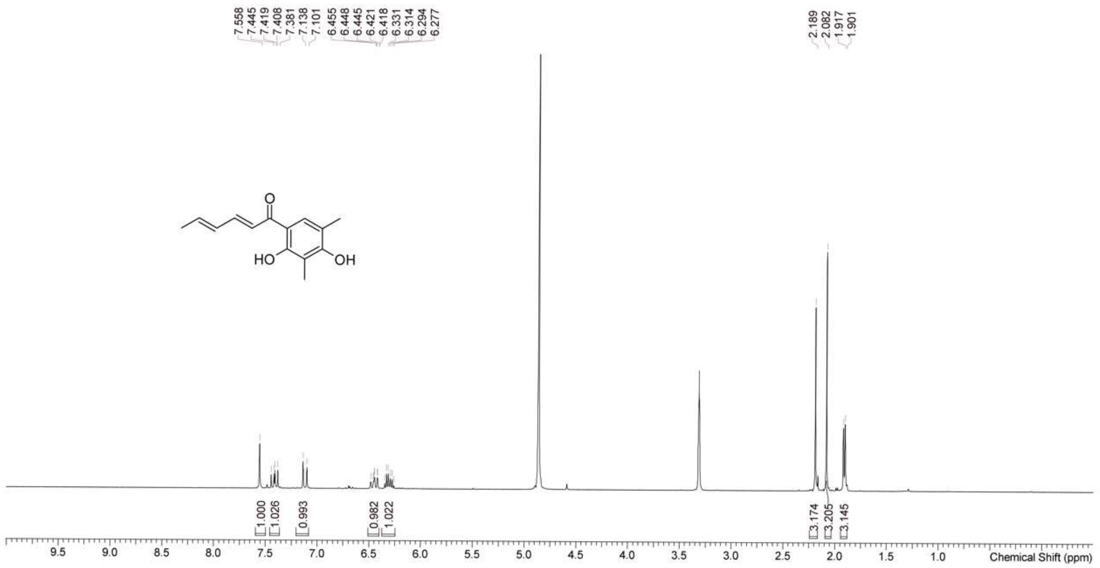

Figure S21. ^{1}H-NMR spectra of sorbicillin. (400 MHz, MeOD) = δ ppm: 7.56 (s, 1H), 7.45-7.38 (m, 1H), 7.14-7.10 (d, 1H), 6.45-6.42 (m, 1H), 6.33-6.27 (m, 1H), 2.19 (s, 3H), 2.08 (s, 3H), 1.92-1.90 (d, 3H).

4. References

(1) Arentshorst, M.; Kooloth Valappil, P.; Mózsik, L.; Regensburg-Tuïnk, T. J. G.; Seekles, S. J.; Tjallinks, G.; Fraaije, M. W.; Visser, J.; Ram, A. F. J. A CRISPR/Cas9-Based Multicopy Integration System for Protein Production in *Aspergillus niger*. *FEBS Journal* **2023**, *290* (21), 5127–5140. https://doi.org/10.1111/febs.16891.

(2) Marillonnet, S.; Grützner, R. Synthetic DNA Assembly Using Golden Gate Cloning and the Hierarchical Modular Cloning Pipeline. *Curr Protoc Mol Biol* **2020**, *130* (1). https://doi.org/10.1002/cpmb.115.

(3) Kunkel, T. A. Rapid and Efficient Site-Specific Mutagenesis without Phenotypic Selection. *Genetics* **1985**, *82*, 488–492.

(4) Peter Macheroux. UV-Visible Spectroscopy as a Tool to Study Flavoproteins. In *Flavoprotein Protocols*; 1999; Vol. 131, pp 1–7.

(5) Bowler, M. W.; Nurizzo, D.; Barrett, R.; Beteva, A.; Bodin, M.; Caserotto, H.; Delagenière, S.; Dobias, F.; Flot, D.; Giraud, T.; Guichard, N.; Guijarro, M.; Lentini, M.; Leonard, G. A.; McSweeney, S.; Oskarsson, M.; Schmidt, W.; Snigirev, A.; Von Stetten, D.; Surr, J.; Svensson, O.; Theveneau, P.; Mueller-Dieckmann, C. MASSIF-1: A Beamline Dedicated to the Fully Automatic Characterization and Data Collection from Crystals of Biological Macromolecules. *J Synchrotron Radiat* **2015**, *22*, 1540–1547. https://doi.org/10.1107/S1600577515016604.

(6) Vonrhein, C.; Flensburg, C.; Keller, P.; Sharff, A.; Smart, O.; Paciorek, W.; Womack, T.; Bricogne, G. Data Processing and Analysis with the *AutoPROC* Toolbox. *Acta Crystallogr D Biol Crystallogr* **2011**, *67* (4), 293–302. https://doi.org/10.1107/S0907444911007773.

(7) McCoy, A. J.; Grosse-Kunstleve, R. W.; Adams, P. D.; Winn, M. D.; Storoni, L. C.; Read, R. J. *Phaser* Crystallographic Software. *J Appl Crystallogr* **2007**, *40* (4), 658–674. https://doi.org/10.1107/S0021889807021206.

(8) Emsley, P.; Cowtan, K. *Coot*: Model-Building Tools for Molecular Graphics. *Acta Crystallogr D Biol Crystallogr* **2004**, *60* (12 I), 2126–2132. https://doi.org/10.1107/S0907444904019158.

(9) Kovalevskiy, O.; Nicholls, R. A.; Long, F.; Carlon, A.; Murshudov, G. N. Overview of Refinement Procedures within *REFMAC* 5: Utilizing Data from Different Sources. *Acta Crystallogr D Struct Biol* **2018**, *74*, 215–227. https://doi.org/10.1107/S2059798318000979.

(10) Potterton, L.; Agirre, J.; Ballard, C.; Cowtan, K.; Dodson, E.; Evans, P. R.; Jenkins, H. T.; Keegan, R.; Krissinel, E.; Stevenson, K.; Lebedev, A.; McNicholas, S. J.; Nicholls, R. A.; Noble, M.; Pannu, N. S.; Roth, C.; Sheldrick, G.; Skubak, P.; Turkenburg, J.; Uski, V.; Von Delft, F.; Waterman, D.; Wilson, K.; Winn, M.; Wojdyr, M. *CCP*4*i*2: The New Graphical User Interface to the *CCP*4 Program Suite. *Acta Crystallogr D Struct Biol* **2018**, *74*, 68–84. https://doi.org/10.1107/S2059798317016035.

(11) Liebschner, D.; Afonine, P. V.; Moriarty, N. W.; Poon, B. K.; Sobolev, O. V.; Terwilliger, T. C.; Adams, P. D. Polder Maps: Improving OMIT Maps by Excluding Bulk Solvent. *Acta Crystallogr D Struct Biol* **2017**, *73* (2), 148–157. https://doi.org/10.1107/S2059798316018210.

(12) Pettersen, E. F.; Goddard, T. D.; Huang, C. C.; Meng, E. C.; Couch, G. S.; Croll, T. I.; Morris, J. H.; Ferrin, T. E. UCSF ChimeraX: Structure Visualization for Researchers, Educators, and Developers. *Protein Science* **2021**, *30* (1), 70–82. https://doi.org/10.1002/pro.3943.

(13) Forneris, F.; Orru, R.; Bonivento, D.; Chiarelli, L. R.; Mattevi, A. *Thermo*FAD, a *Thermofluor*®-Adapted Flavin *ad hoc* Detection System for Protein Folding and Ligand Binding. *FEBS Journal* **2009**, *276* (10), 2833–2840. https://doi.org/10.1111/j.1742-4658.2009.07006.x.

(14) Copeland, R. A. *Enzymes: A Practical Introduction to Structure, Mechanism, and Data Analysis*; Wiley-VCH: New York, 2000.
